# Fascin1-Dependent Filopodia are Required for Directional Migration of a Subset of Neural Crest Cells

**DOI:** 10.1371/journal.pgen.1004946

**Published:** 2015-01-21

**Authors:** Elena F. Boer, Elizabeth D. Howell, Thomas F. Schilling, Cicely A. Jette, Rodney A. Stewart

**Affiliations:** 1 Department of Oncological Sciences, Huntsman Cancer Institute, University of Utah, Salt Lake City, Utah, United States of America; 2 Department of Developmental and Cell Biology, University of California, Irvine, Irvine, California, United States of America; California Institute of Technology, UNITED STATES

## Abstract

Directional migration of neural crest (NC) cells is essential for patterning the vertebrate embryo, including the craniofacial skeleton. Extensive filopodial protrusions in NC cells are thought to sense chemo-attractive/repulsive signals that provide directionality. To test this hypothesis, we generated null mutations in zebrafish *fascin1a (fscn1a)*, which encodes an actin-bundling protein required for filopodia formation. Homozygous *fscn1a* zygotic null mutants have normal NC filopodia due to unexpected stability of maternal Fscn1a protein throughout NC development and into juvenile stages. In contrast, maternal/zygotic *fscn1a* null mutant embryos (*fscn1a MZ*) have severe loss of NC filopodia. However, only a subset of NC streams display migration defects, associated with selective loss of craniofacial elements and peripheral neurons. We also show that *fscn1a*-dependent NC migration functions through *cxcr4a/cxcl12b* chemokine signaling to ensure the fidelity of directional cell migration. These data show that *fscn1a*-dependent filopodia are required in a subset of NC cells to promote cell migration and NC derivative formation, and that perdurance of long-lived maternal proteins can mask essential zygotic gene functions during NC development.

## Author Summary

During vertebrate embryogenesis, neural crest (NC) cells migrate extensively along stereotypical migration routes and differentiate into diverse derivatives, including the craniofacial skeleton and peripheral nervous system. While defects in NC migration underlie many human birth defects and may be coopted during cancer metastasis, the genetic pathways controlling directional NC migration remain incompletely understood. Filopodia protrusions are thought to act as “cellular antennae” that explore the environment for directional cues to ensure NC cells reach their correct location. To test this idea, we generated zebrafish *fascin1a (fscn1a)* mutants that have severe loss of filopodia. Surprisingly, we found that most NC cells migrate to their correct locations without robust filopodial protrusions. We found that *fscn1a* embryos have directional migration defects in a subset of NC cells, resulting in loss of specific craniofacial elements and peripheral neurons. Interestingly, these defects were only observed in ∼20% of *fscn1a* embryos, but were significantly enhanced by partial loss of the chemokine receptor Cxcr4a or disruption of the localized expression of its ligand Cxcl12b. Our data show that subsets of skeletal and neurogenic NC cells require filopodia to migrate and that *fscn1a*-dependent filopodia cooperate with chemokine signaling to promote directional migration of a subset of NC cells.

## Introduction

Directional cell migration is used by a variety of cell types throughout embryonic development, wound healing, and cancer metastasis [1]. Distinct chemo-attractive/repulsive mechanisms, involving cell surface receptors and their secreted ligands, are common mechanisms that guide cells to their correct locations. Filopodial protrusions at the leading edges of migrating cells are thought to facilitate directional migration by acting as cellular antennae that actively monitor the distribution of guidance molecules in the environment [2,3]. Indeed, disrupting filopodia dynamics reduces the directional efficiency of migrating cells in culture [4–6] and selectively restricts cell migration in hematopoietic and neuronal lineages *in vivo* [7–9]. In contrast, recent studies have shown filopodia are dispensable for endothelial tip cell guidance during angiogenesis [10,11]. These studies suggest that filopodia have unique functions in a subset of cell types and/or may act redundantly with other guidance mechanisms to promote cell migration *in vivo*, although the selective nature of these mechanisms remains largely unknown.

Neural crest (NC) development is a dramatic example of directional cell migration in vertebrate embryos. Following delamination from the neural tube, multipotent NC cells migrate extensively throughout the embryo along stereotypical pathways to form a variety of cell types [12]. Cranial NC cells that form the craniofacial skeleton migrate as collective cell populations from the developing midbrain and hindbrain into pharyngeal arches, while other cranial NC cells migrate less collectively to generate peripheral neurons and glia of the cranial nerves and pigment cells, among other derivatives [13]. Vagal/cardiac and trunk NC cells initially migrate along a medial pathway to form ganglia of the peripheral nervous system, as well as connective tissue of the heart and outflow tract. At later stages, single trunk NC cells migrate along a lateral pathway to produce pigment cells in the skin [12]. Thus, the mode of NC migration (collective versus individual) and pathway selection (medial versus lateral) correlates with axial position, time of delamination and ultimate fate. Coordination of these migration paths is essential for patterning the vertebrate body plan and defects in NC migration mechanisms are associated with congenital defects and pediatric cancers, collectively referred to as neurocristopathies [14].

Premigratory NC cells display a polarized morphology associated with Rho GTPase activation in retracting NC cell tails and Rac1 GTPase activation in the direction of cell migration, which promotes F-actin polymerization and formation of cell membrane protrusions, including lamellipodia and filopodia [15–17]. Filopodial protrusions are dynamic cellular processes; *in vivo* imaging of migrating NC cells shows that filopodia are rapidly generated in the direction of chemo-attractive cues but collapse when exposed to repulsive cues [18]. In addition, NC protrusions are evident during contact inhibition of locomotion and coattraction, behaviors associated with cell-cell repulsion and adhesion that are proposed to drive the overall direction of some collective NC streams [19,20]. Most, if not all, of these mechanisms implicate dynamic filopodia extension and retraction as essential mediators of the cellular behaviors observed during directional NC migration, however this has not been directly tested.

Fascin1 (*Fscn1*) is required for F-actin bundling, filopodia formation and migration in a variety of metazoan cell types [21]. *Fscn1* is highly upregulated in aggressive tumors, where it promotes cell migration when overexpressed and blocks migration and invasion when inhibited [22,23]. *In vivo* studies in *Drosophila* and mouse have demonstrated requirements for *Fscn1* during individual-cell migration of hemocytes, neuroblasts, dendritic cells and melanoblasts [8,9,24,25]. However, the role of *Fscn1* in early vertebrate embryogenesis and collective cell migration *in vivo* remains unknown, due in part to the fact that it is not known if the first intron retroviral insertion allele of *Fscn1* affects mRNA or protein expression in the early mouse embryo [25,26]. In addition, the molecular mechanism(s) by which *Fscn1* promotes cell migration *in vivo* is still poorly understood, likely due to redundancy with other directional cell migration mechanisms.

To determine if Fscn1-dependent filopodia are required for NC cell migration, we generated TALEN-induced null mutations in zebrafish *fscn1a*, the only *fascin* gene expressed in zebrafish NC cells. Surprisingly, homozygous *fscn1a* null mutants have no defects in NC filopodia formation and are viable and fertile. Analysis of protein levels in oocytes and zygotic null *fscn1a* mutants reveals that Fscn1a protein is maternally deposited and remarkably stable (up to 10 days post fertilization), lasting throughout embryonic development and organogenesis and masking potential zygotic functions of *fscn1a* in NC migration. In contrast, maternal/zygotic *fscn1a* (*fscn1a MZ*) mutant embryos show severe defects in the number, length and dynamics of NC filopodia and selective defects in migration of a subset of cranial NC streams. The *fscn1a* null NC phenotypes are partially penetrant and often asymmetric, leading to the loss of single cartilage elements on one side of the face. *fscn1a MZ* mutants also have selective loss of NC-derived peripheral sympathetic and enteric neurons, but not dorsal root ganglia. Importantly, while depletion of residual filopodia in *fscn1a* null mutants with the F-actin polymerizing inhibitor Latrunculin B enhanced *fscn1a*-dependent NC derivative defects, most NC cells still migrated normally, showing that filopodia are largely dispensable for NC migration. We also demonstrate that *fscn1a* controls directional migration of the first cranial NC stream through interactions with the chemokine receptor *chemokine (C-X-C motif) receptor 4a (cxcr4a)* and its ligand *chemokine (C-X-C motif) ligand 12b* (*cxcl12b, or sdf1b)*. Together, these data show that perdurance of stable maternal proteins can mask essential zygotic gene functions in organogenesis, and demonstrate differential requirements for filopodia in directional NC migration and subsequent NC derivative formation.

## Results

### *fscn1a* expression in migrating NC cells requires *tfap2a*

**Figure 1:**
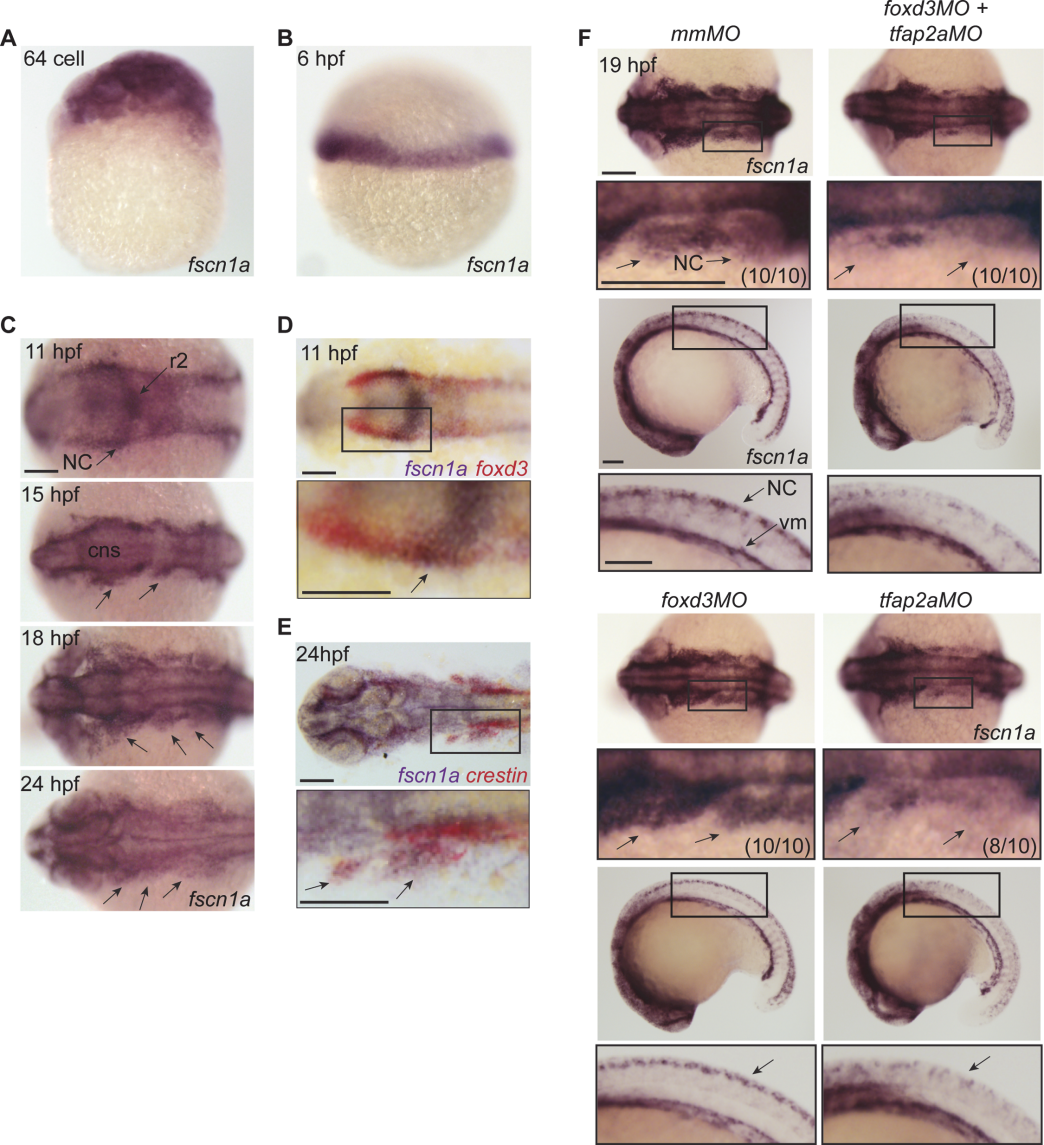
*fscn1a* mRNA expression in zebrafish NC is regulated by *tfap2a*. (A) Whole-mount ISH of maternal *fscn1a* mRNA at the 64-cell stage. (B) *fscn1a* mRNA expression at 6 hours post fertilization (hpf) at the gastrula margin. (C) Dorsal cranial views of *fscn1a* expression in 11, 15, 18, and 24 hpf embryos. 11 hpf embryos show expression in the brain, particularly rhombomere 2 (r2), and neural crest (NC) (top panel). In 15 and 18 hpf embryos, *fscn1a* is expressed in the brain (CNS) and cranial NC streams (middle panels, arrows). In 24 hpf embryos, *fscn1a* is expressed in the brain and pharyngeal arches (bottom panel, arrows). (D) In 11 hpf embryos, *fscn1a* mRNA is co-expressed with *foxd3* at the neural plate border, highlighted in a magnified view (bottom panel). (E) *fscn1a* mRNA is co-expressed with *crestin* in cranial NC streams at 24 hpf, highlighted in a magnified view (bottom panel). (F) Dorsal cranial (top panels) and lateral (bottom panels) views of *fscn1a* expression in 19 hpf embryos injected with mismatch MO (*mmMO*), *foxd3MO* and *tfap2aMO* (*foxd3MO+tfap2aMO*), *foxd3MO*, or *tfap2aMO*. Magnified views of boxed regions are below each panel. Numbers in parentheses indicate proportion of embryos with phenotype. Scale bars = 100 μm.

Fascin proteins are required for filopodia formation [21]. To determine which fascins in zebrafish might regulate filopodia during NC migration, we evaluated the spatiotemporal expression of *fascin1a* (*fscn1a*) and *fascin1b* (*fscn1b*) by whole-mount RNA *in situ* hybridization (ISH). Only *fscn1a* mRNA was detected in the NC (Fig. 1), whereas *fscn1b* expression was restricted to the telencephalon from 36 hpf onwards (S1 Fig.). Maternal *fscn1a* mRNA is ubiquitously expressed (Fig. 1A), but by 6 hpf (50% epiboly) is restricted to the involuting blastoderm margin (Fig. 1B). At 11 hpf, *fscn1a* is expressed in rhombomere 2 (r2) of the hindbrain and at lower levels in adjacent neural tube and along the neural plate border (Fig. 1C), where its expression partially overlaps with *foxd3* in NC (Fig. 1D) [27]. During cranial NC migration (12–24 hpf), *fscn1a* is expressed in migrating NC streams (Fig. 1C) and co-localizes with the NC marker *crestin* (Fig. 1E) [28]. In 18 and 24 hpf embryos, *fscn1a* is expressed in spinal cord neurons and *crestin^+^* trunk NC cells, as well as in somites and vasculogenic mesoderm (S2 Fig.).

In zebrafish, loss of *foxd3* and *tfap2a* causes a complete absence of NC cells [29,30]. To confirm *fscn1a* expression in NC cells, we injected antisense morpholino (MO) oligonucleotides targeting *foxd3* (*foxd3MO*) and/or *tfap2a* (*tfap2aMO*) and performed ISH to detect *fscn1a* mRNA (Fig. 1F). As expected, ablation of NC by co-injecting *tfap2aMO and foxd3MO* caused loss of *fscn1a* expression only in the dorsal neural tube, but not in other cell types in the embryo such as vasculogenic mesoderm, consistent with the hypothesis that *fscn1a* is expressed in NC. Injection of *foxd3MO* or *tfap2aMO* alone showed that the NC-specific expression pattern of *fscn1a* depends on *tfap2a* (Fig. 1F).

### Maternal Fscn1a protein persists in *fscn1a* mutants throughout embryonic development

Having established *fscn1a* expression in the NC, we next wanted to determine if *fscn1a* was required for NC filopodia formation and cell migration. We generated several null alleles of *fscn1a* using first exon-targeted TALENs (S3A-S3C Fig.). The *fscn1a^Δ7^, fscn1a^Δ10^, fscn1a^+17^* alleles all harbor frame-shift deletions in exon 1 that generate premature stop codons immediately upstream of the highly conserved F-actin binding site required for actin bundling (S3C-S3D Fig.) [31].

**Figure 2:**
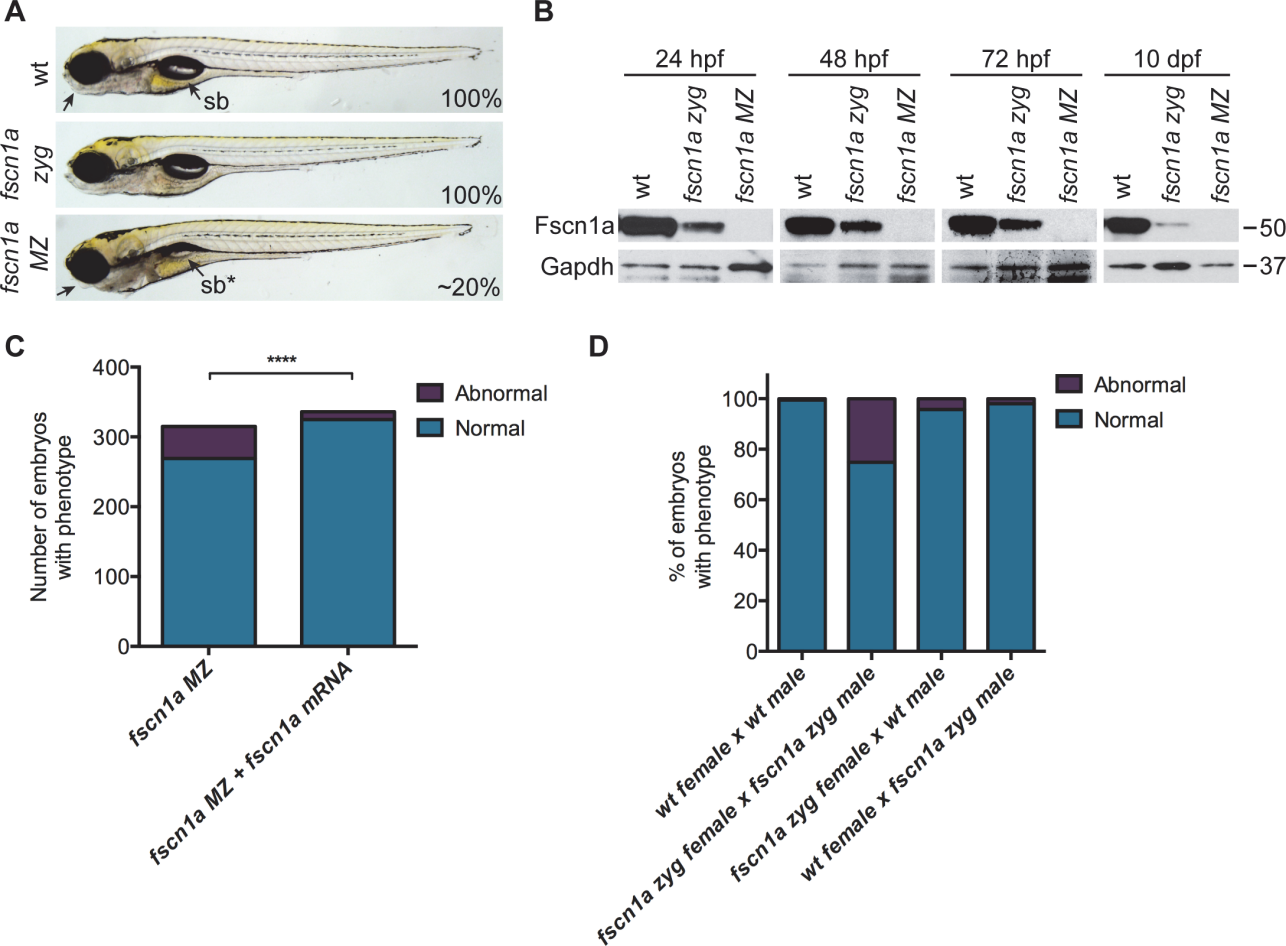
Maternal Fscn1a protein persists throughout embryogenesis and masks morphological defects in *fscn1a* zygotic mutants. (A) Brightfield images of 5 dpf wild type (wt), zygotic *fscn1a* (*fscn1a zyg*), and maternal/zygotic *fscn1a* (*fscn1a MZ*) embryos. Arrow highlights craniofacial cartilage, sb = swim bladder. Number indicates percentage of embryos with phenotype. (B) Immunoblot showing Fscn1a protein levels at 24, 48, 72 hpf and 10 dpf in wt, *fscn1a zyg*, and *fscn1a MZ* mutant embryos. Numbers on right denote molecular mass markers. (C) Quantitation of uninjected or *fscn1a* mRNA-injected *fscn1a MZ* embryos with normal vs. abnormal craniofacial cartilages at 5 dpf (n = 315 *fscn1a MZ* embryos, 336 *fscn1a MZ* + *fscn1a* mRNA embryos, ****p<0.0001). (D) Quantitation of embryos with normal vs. abnormal craniofacial cartilages at 5 dpf (n = 201 wt female × wt male, 119 *fscn1a zyg* female × *fscn1a zyg* male, 115 *fscn1a zyg* female × wt male, 151 wt female × *fscn1a zyg* male).

We incrossed *fscn1a* heterozygous adults to generate homozygous *fscn1a* zygotic (zyg) mutant embryos, which were viable with no obvious morphological abnormalities (Fig. 2A). As *fscn1a* is expressed maternally (Fig. 1A, S4 Fig.), we hypothesized that maternally deposited Fscn1a protein may mask essential early functions for zygotic *fscn1a* during development. We analyzed Fscn1a protein levels in *fscn1a zyg* embryos at 24, 48 and 72 hpf, and 10 days post fertilization (dpf) by immunoblot analysis (Fig. 2B). Unexpectedly, when NC cells are actively migrating (12–72 hpf), maternally deposited Fscn1a protein is present in *fscn1a zyg* embryos at levels comparable to wild type siblings and persists at moderate levels through 10 dpf (Fig. 2B). Thus, maternal Fscn1a protein lasts throughout all organogenesis stages, potentially masking roles for zygotic *fscn1a* in NC development.

To reveal potential embryonic roles for zygotic *fscn1a* expression, we incrossed homozygous *fscn1a zyg* animals to generate maternal/zygotic *fscn1a* mutant embryos (*fscn1a MZ*, S3B Fig.). *fscn1a MZ* oocytes were viable and, following fertilization, embryos showed no obvious defects in cleavage or gastrulation. However, at 5 dpf *fscn1a MZ* embryos displayed gross morphological abnormalities in the jaw, heart, and swim bladder with partial penetrance ranging from 0–50%, with an average of ∼20% (Fig. 2A). The remaining *fscn1a MZ* embryos were morphologically normal and survived to adulthood, suggesting possible redundancy with other *fscn* zebrafish paralogs. Therefore, we analyzed the temporal expression of *fscn1b, fscn2a*, and *fscn2b* in *fscn1a MZ* mutants and observed no aberrant expression (e.g. upregulation) that might explain the partial penetrance (S5 Fig.). In addition, MO knockdown of all other *fscn* paralogs (which are not maternally expressed) did not affect the penetrance of the *fscn1a MZ* mutant phenotypes (S6 Fig.). Importantly, the *fscn1a MZ* morphological defects were fully rescued by injecting *fscn1a* mRNA (Fig. 2C). Combined, these data show that *fscn1a* function is essential for normal embryonic development, but is partially penetrant.

To determine the contribution of paternal (zygotic only) *fscn1a* expression to embryonic development, homozygous *fscn1a zyg* females were bred to wild type males. 96% of embryos derived from this cross did not display obvious morphological defects (Fig. 2D), demonstrating that paternal (zygotic) *fscn1a* expression is sufficient to rescue *fscn1a MZ* morphological defects, and that perdurance of maternal Fscn1a protein masks essential zygotic functions of *fscn1a* in embryogenesis.

### *fscn1a* is required for filopodia formation and dynamics in NC cells

**Figure 3:**
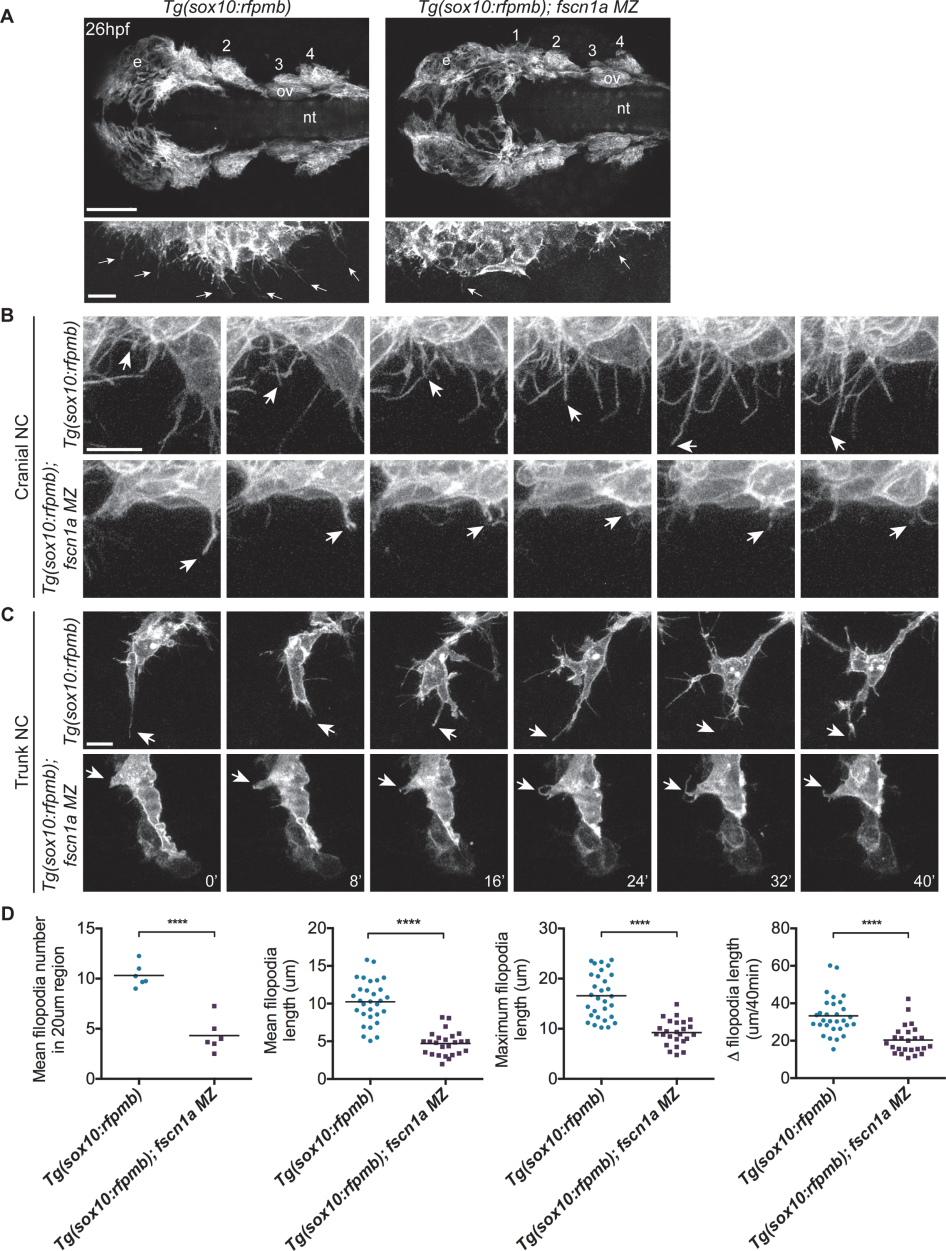
*fscn1a* is required for filopodia formation in NC cells. (A) Dorsal cranial views of cranial NC streams in 26 hpf *Tg(sox10:rfpmb)* and *Tg(sox10:rfpmb); fscn1a MZ* embryos, anterior is to the left. Numbers correspond to pharyngeal arches, e = eye, ov = otic vesicle, nt = neural tube, scale bar = 100μm. Leading edge of NC stream 1 is enlarged in right panel, scale bar = 10 μm. Arrows highlight filopodia at leading edge of NC stream 1. (B-C) Time lapse confocal images of filopodia at the leading edge of NC stream 2 (B) or of leading cells in trunk NC streams (C) in live *Tg(sox10:rfpmb)* and *Tg(sox10:rfpmb)*; *fscn1a MZ* embryos at 26 hpf. Arrows highlight tips of single filopodia throughout the time lapses. Scale bar = 10 μm. (D) Quantitation of filopodia number, mean and maximum filopodia length, and change in filopodia length in *Tg(sox10:rfpmb)* and *Tg(sox10:rfpmb); fscn1a MZ* cranial NC (n = 6 embryos and 30 filopodia each for *Tg(sox10:rfpmb)* and *Tg(sox10:rfpmb); fscn1a MZ*, ****p<0.0001).

To determine if *fscn1a* is required for filopodia formation in NC cells, we analyzed *Tg(sox10:rfpmb)* and *Tg(sox10:rfpmb); fscn1a MZ* embryos in which *sox10*-expressing NC cells are labeled with a membrane-bound RFP, enabling visualization of membrane protrusions [32]. In 26 hpf wild type embryos, dynamic filopodial projections were visible at the leading edge of all cranial and trunk NC streams (Fig. 3A-C and S1 Movie). In contrast, filopodia at the leading edge of *fscn1a MZ* NC streams were significantly reduced in number, length, and change in length over time (Fig. 3A-D and S2 Movie). In addition, compared to wild type NC cells, the morphology of *fscn1a MZ* NC cells appeared flatter and less polarized (S1–S2 Movies). This phenotype was particularly evident in small cohorts of migrating trunk NC cells (Fig. 3C). Importantly, the penetrance of filopodia defects in *fscn1a MZ* embryos was 100% (n = 6 cranial, n = 6 trunk), in contrast to the partially penetrant morphological defects (Fig. 2A). These results show that, similar to other organisms, zebrafish *fscn1a* has an essential role in generating robust filopodia *in vivo*.

### *fscn1a* is required for migration of a subset of cranial NC streams

**Figure 4:**
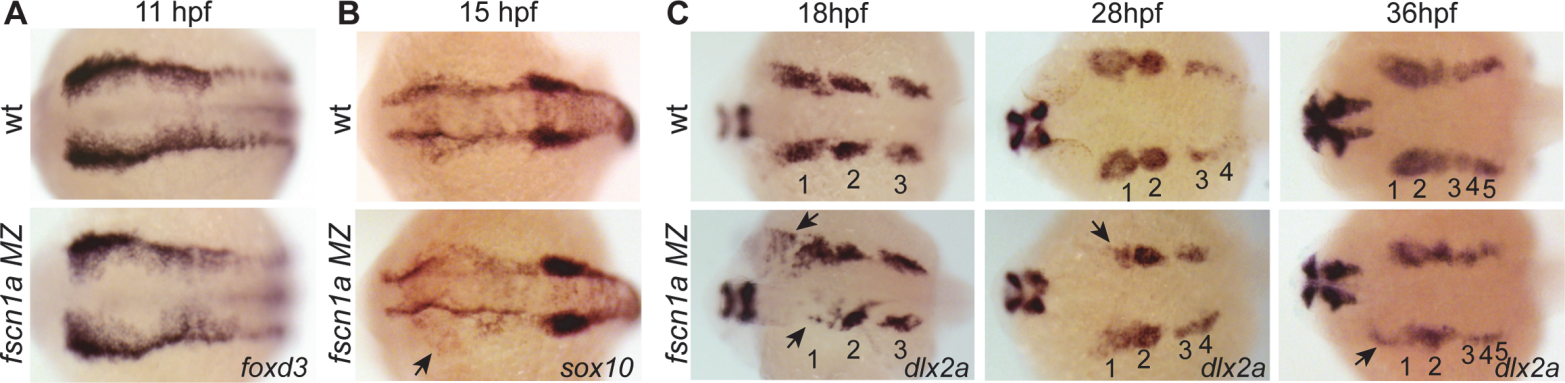
*fscn1a* is required for collective migration of the first NC stream. Dorsal cranial views of wild type and *fscn1a MZ* embryos analyzed by RNA *in situ* hybridization for (A) *foxd3* mRNA at 11 hpf, (B) *sox10* mRNA at 15 hpf, showing asymmetric dispersion of NC cells in *fscn1a MZ* (arrow), and (C) *dlx2a* mRNA at 18, 28 and 36 hpf. Numbers correspond to pharyngeal arches, arrows indicate abnormal NC migration phenotypes observed in *dlx2a+* NC cells in *fscn1a MZ* embryos. Anterior is left in all panels. Phenotypes shown in *fscn1a MZ* panels were observed in 100% (A) or ∼20% (B-C) of embryos examined (n>200).

We next asked if *fscn1a*-dependent filopodia were required for NC induction and migration. Examination of *foxd3* mRNA expression by ISH at 11 hpf in *fscn1a MZ* embryos (Fig. 4A) revealed no difference between *fscn1a MZ* and wild type siblings, suggesting *fscn1a* is not required for NC induction. We next analyzed markers of NC migration. To examine early cranial NC migration, the expression pattern of *sox10* mRNA was analyzed in wild type and *fscn1a MZ* embryos by ISH at 15 hpf. At this time point, ∼20% of *fscn1a MZ* embryos displayed abnormal NC migration, including a dispersion of the most anterior *sox10+* NC cells over the yolk, suggesting a primary defect in the directional migration of the first cranial NC stream (Fig. 4B). To confirm this, we examined the expression pattern of *dlx2a*, which is expressed in migratory NC cells that contribute to the pharyngeal arches, at 18, 28 and 36 hpf. At all time points, ∼20% of *fscn1a MZ* embryos showed loss or reduction of *dlx2a* specifically in the mandibular arch, suggesting that a subset of cranial NC cells fail to migrate to the pharyngeal arches (Fig. 4C). In addition, *dlx2a* ^+^ NC cells were observed outside of the normal stream boundaries in ∼20% of *fscn1a MZ* embryos, often in an asymmetric manner. At 36 hpf, *dlx2a+* NC cells condense within the mandibular arch primordia in wild type embryos. In contrast, *dlx2a+* NC cells in ∼20% of *fscn1a MZ* embryos remained more dispersed over the yolk and anterior to the normal location of the mandibular primordium (Fig. 4C), showing that *fscn1a* function is required in a subset of NC streams to direct them to the correct location.

To determine if the *fscn1a*-dependent defects in directional NC migration were due to abnormal cell migration behaviors, we examined cranial NC streams in *Tg(sox10:rfpmb)* and *Tg(sox10:rfpmb); fscn1a MZ* embryos by confocal time-lapse imaging. In wild type embryos we observed that NC streams destined for the pharyngeal arches are tightly associated and dynamic cellular protrusions are predominant in cells at the leading edges and sides of the streams (S3 Movie). Overall, we observed no obvious differences in individual cell behaviors at the leading edges of NC streams between wild type and *fscn1a MZ* cranial NC streams (n = 20 embryos, S3–S4 Movies), although occasionally we observed a cell at the leading edge move away from the stream in the *fscn1a MZ* mutants (S4 Movie). These data, together with data from Fig. 4, suggest that *fscn1a*-dependent filopodia are not required for individual cell migration behaviors, like contact-dependent adhesion, but instead are required for directional migration of a subset of collective NC streams.

### A subset of NC derivatives are lost in *fscn1a MZ* mutants

**Figure 5:**
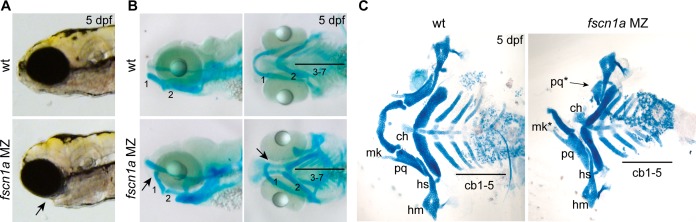
*fscn1a* is required for development of NC-derived craniofacial cartilage. (A) Lateral views of wild type (wt) and *fscn1a MZ* 5 dpf embryos. Arrow indicates reduced jaw in *fscn1a MZ* embryos. (B) Lateral (left) and ventral (right) views of Alcian blue-stained wt and *fscn1a MZ* 5 dpf embryos. Numbers correspond to pharyngeal arches. Arrow indicates missing palatoquadrate in *fscn1a MZ* embryos. (C) Ventral view of dissected Alcian blue-stained pharyngeal skeletons from wt and *fscn1a MZ* 5 dpf embryos. ch = ceratohyal, cb = ceratobranchial, hm = hyomandibular, hs = hyosymplectic, mk = Meckel’s cartilage, pq = palatoquadrate.

We next sought to understand the developmental consequence of *fscn1a*-dependent filopodia loss and aberrant cell migration behaviors. NC cells give rise to many cell types, including the craniofacial skeleton, heart outflow tract, neurons and glia of the peripheral nervous system, and pigment-producing melanophores [12]. The most prominent phenotype in *fscn1a MZ* embryos is abnormal craniofacial morphology in approximately 20% of embryos (Fig. 5A). The pharyngeal skeleton is derived from cranial NC cells that migrate as three distinct streams from the dorsal region of the hindbrain to the mandibular (stream 1), hyoid (stream 2), and branchial (stream 3) arches, with stream 3 in zebrafish further subdividing into five separate streams to form the gill arches [13,33]. In *fscn1a MZ* embryos stained with Alcian blue, a specific loss of elements derived from the mandibular arch was evident in ∼20% of 5 dpf embryos (Fig. 5B). Dissection of Alcian blue-stained pharyngeal cartilages, as well as live imaging of *sox10*+ pharyngeal cartilages in 5 dpf *Tg(sox10:rfpmb); fscn1a MZ* embryos, revealed symmetric or asymmetric loss of first stream derivatives, including Meckel’s and palatoquadrate cartilages (Fig. 5C, S7 Fig.). Among the ∼20% of *fscn1a MZ* embryos displaying abnormal craniofacial morphology, the distribution of symmetric, left-sided asymmetric, and right-sided asymmetric defects was stochastic (S7 Fig.). Defects in hyoid and branchial arch elements were never observed, consistent with normal migration of these NC streams into the pharyngeal arches. These results show that *fscn1a*-dependent filopodia are required for the normal development of a subset of NC-derived craniofacial cartilages, consistent with the selective defects we observed in cranial NC migration (Fig. 4B,C).

**Figure 6:**
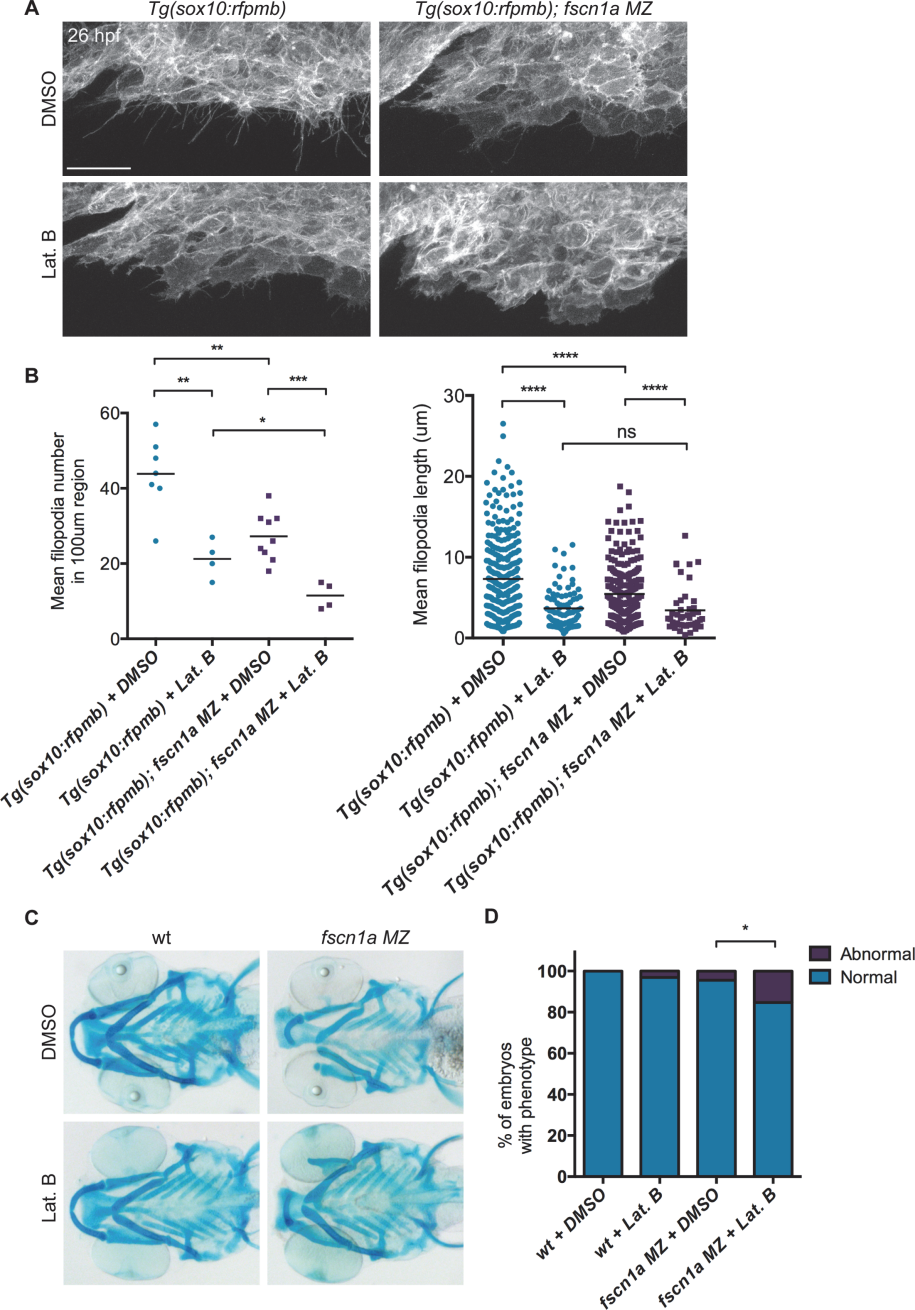
Depletion of *fscn1a*-independent NC cell filopodia by low concentration Latrunculin B treatment does not enhance the severity of *fscn1a MZ* craniofacial defects. (A) Lateral views of leading edge of cranial NC stream 1 in 26 hpf DMSO or Lat. B-treated *Tg(sox10:rfpmb)* or *Tg(sox10:rfpmb); fscn1a MZ* embryos. Scale bar = 25 μm. (B) Quantitation of filopodia number and filopodia length in DMSO or Lat. B-treated *Tg(sox10:rfpmb)* and *Tg(sox10:rfpmb); fscn1a MZ* embryos (n = 9 *Tg(sox10:rfpmb)* + DMSO, n = 10 *Tg(sox10:rfpmb)* + Lat. B, n = 10 *Tg(sox10:rfpmb); fscn1a MZ* + DMSO, n = 10 *Tg(sox10:rfpmb); fscn1a MZ* + Lat. B, *p = 0.02, **p<0.005, ***p<0.001, ****p<0.0001, ns = not significant). (C) Representative ventral views of 5 dpf DMSO or Lat. B-treated wild type (wt) and *fscn1a MZ* embryos stained with Alcian blue. (D) Quantitation of percentage of embryos with normal or abnormal craniofacial development at 5 dpf. Numbers above columns indicate number of embryos included in analysis (n = 83 wt + DMSO, 93 wt + Lat. B, n = 86 *fscn1a MZ* + DMSO, n = 72 *fscn1a MZ* + Lat. B, *p = 0.02).

*fscn1a* null mutants do not completely lack filopodia, suggesting that the shorter, less abundant residual filopodia in these mutants still function to promote NC migration. To test this, we treated embryos with the F-actin polymerization inhibitor Latrunculin B (Lat. B), which at low doses (80 ng/ml) has previously been shown to selectively inhibit filopodia formation but not other F-actin structures (lamellipodia, cortical actin) in zebrafish endothelial cells [10]. At 26 hpf, wild type *Tg(sox10:rfpmb)* embryos treated with low doses of Lat. B displayed a severe reduction in the number and length of filopodia at the leading edge of cranial NC streams (Fig. 6A,B). Compared to DMSO-treated *Tg(sox10:rfpmb); fscn1a MZ* embryos, low dose Lat. B-treated *Tg(sox10:rfpmb); fscn1a MZ* embryos displayed a further reduction in NC cell filopodia number and length at 26 hpf, presumably through effects on residual *fscn1a*-independent filopodia (Fig. 6A,B). Despite reducing filopodia, Lat. B did not disrupt the development of NC-derived craniofacial elements in wild type embryos (Fig. 6C,D). Treatment of *fscn1a MZ* embryos with low dose Lat. B had a modest but significant effect on the penetrance of *fscn1a*-dependent craniofacial defects, but did not result in more severe phenotypes (e.g. loss of posterior NC-derived craniofacial cartilages) (Fig. 6C,D). In all, these data suggest that the selective defects in NC migration observed in *fscn1a MZ* mutant embryos are unlikely to be due to residual *fscn1a*-independent filopodia.

The endoderm of the pharyngeal pouches plays important roles in patterning and differentiation of skeletogenic NC cells within the arches [34]. To determine if defects in pharyngeal pouch development contribute to the migration and craniofacial phenotypes observed in *fscn1a MZ* embryos, we examined pharyngeal endoderm at 30 hpf in wild type and *fscn1a MZ* embryos by whole-mount ISH but found no obvious defects in patterning of *nkx2.3+* pharyngeal endoderm (S8 Fig.). These results are consistent with the craniofacial defects in *fscn1a MZ* embryos resulting from abnormal NC cell migration in the mandibular stream, and supported by the observation that abnormal migration of *sox10*-positive NC cells is the first observed defect in *fscn1a MZ* embryos (Fig. 4B), which occurs approximately 4 hours before endodermal pouches are formed.

**Figure 7:**
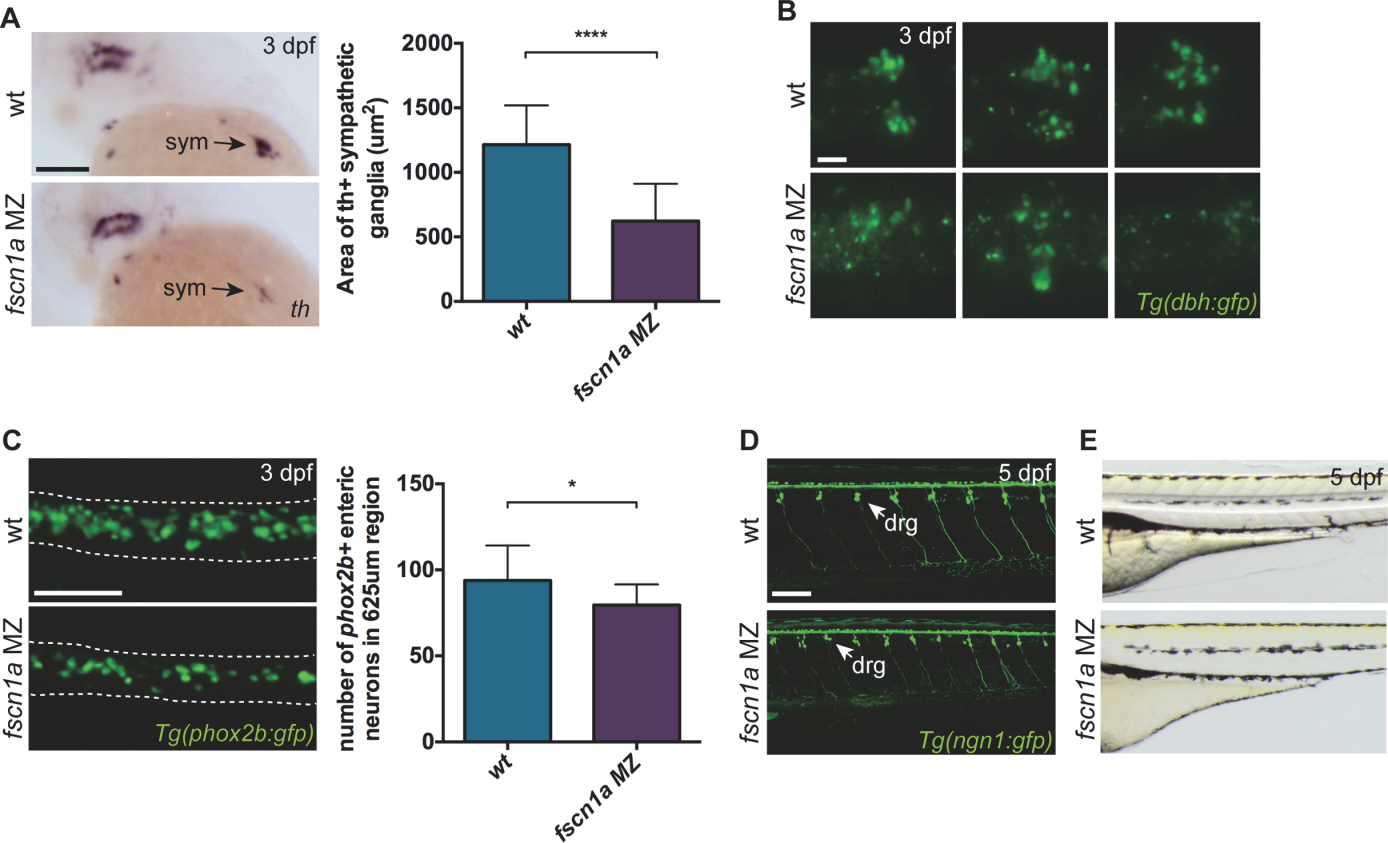
*fscn1a* is required for development of a subset of NC derivatives. (A) Lateral views of 3 dpf wt and *fscn1a MZ* embryos stained for *th*. sym = sympathetic ganglia, scale bar = 100 μm. Mean area of *th+* sympathetic ganglia is plotted in graph on right. (n = 25 wt, 24 *fscn1a MZ*, ****p<0.0001). (B) Representative images of *dbh:gfp+* sympathetic ganglia in 3 dpf wt and *fscn1a MZ* embryos. Dorsal views, anterior to the left. Scale bar = 25 μm. (C) Representative images of *phox2b:gfp+* enteric neurons in 3 dpf wt and *fscn1a MZ* embryos. Dotted lines outline gut. Scale bar = 100 μm. Number of *phox2b:gfp+* enteric neurons in 625 μm region of gut is plotted in graph on right (n = 15 wt, 13 *fscn1 MZ*, *p = 0.03). (D) *ngn1:gfp+* dorsal root ganglia (drg) in 5 dpf wt and *fscn1a MZ* embryos. Scale bar = 100 μm. (E) Lateral views showing melanophores in 5 dpf wt and *fscn1a MZ* embryos.

To determine if NC-derived peripheral neurons derived from the vagal NC stream are present in *fscn1a MZ* embryos, we examined the expression of *tyrosine hydroxylase* (*th*) and dopamine β-hydroxylase (*dbh*), markers of noradrenergic and dopaminergic neurons. At 3 dpf, NC-derived *th* ^+^ sympathetic neurons were severely reduced in ∼20% of *fscn1a MZ* embryos (Fig. 7A). Visualization of sympathetic ganglia in 3 dpf *Tg(dbh:gfp)* embryos confirmed a reduction in *dbh+* sympathetic neurons and revealed a failure of existing *dbh+* sympathetic neurons to condense into ganglia (Fig. 7B). We also analyzed the development of enteric neurons, which are derived from vagal NC cells. At 3 dpf, ∼20% of *fscn1a MZ* embryos displayed a significant reduction in *phox2b+* enteric neurons relative to wild type embryos (Fig. 7C). Interestingly, formation of trunk NC-derived dorsal root ganglia (DRG) was unaffected in 5 dpf *fscn1a MZ* embryos (Fig. 7D). Melanophore pigment cells were also unaffected in 5 dpf *fscn1a MZ* embryos (Fig. 7E). In all, these results show that *fscn1a* is required for the development of a subset of NC lineages, with the most severe defects observed in derivatives of the cranial/vagal NC, which migrate collectively.

### *fscn1a* cooperates with *cxcr4a/cxcl12b* to promote directional migration of cranial NC cells

**Figure 8:**
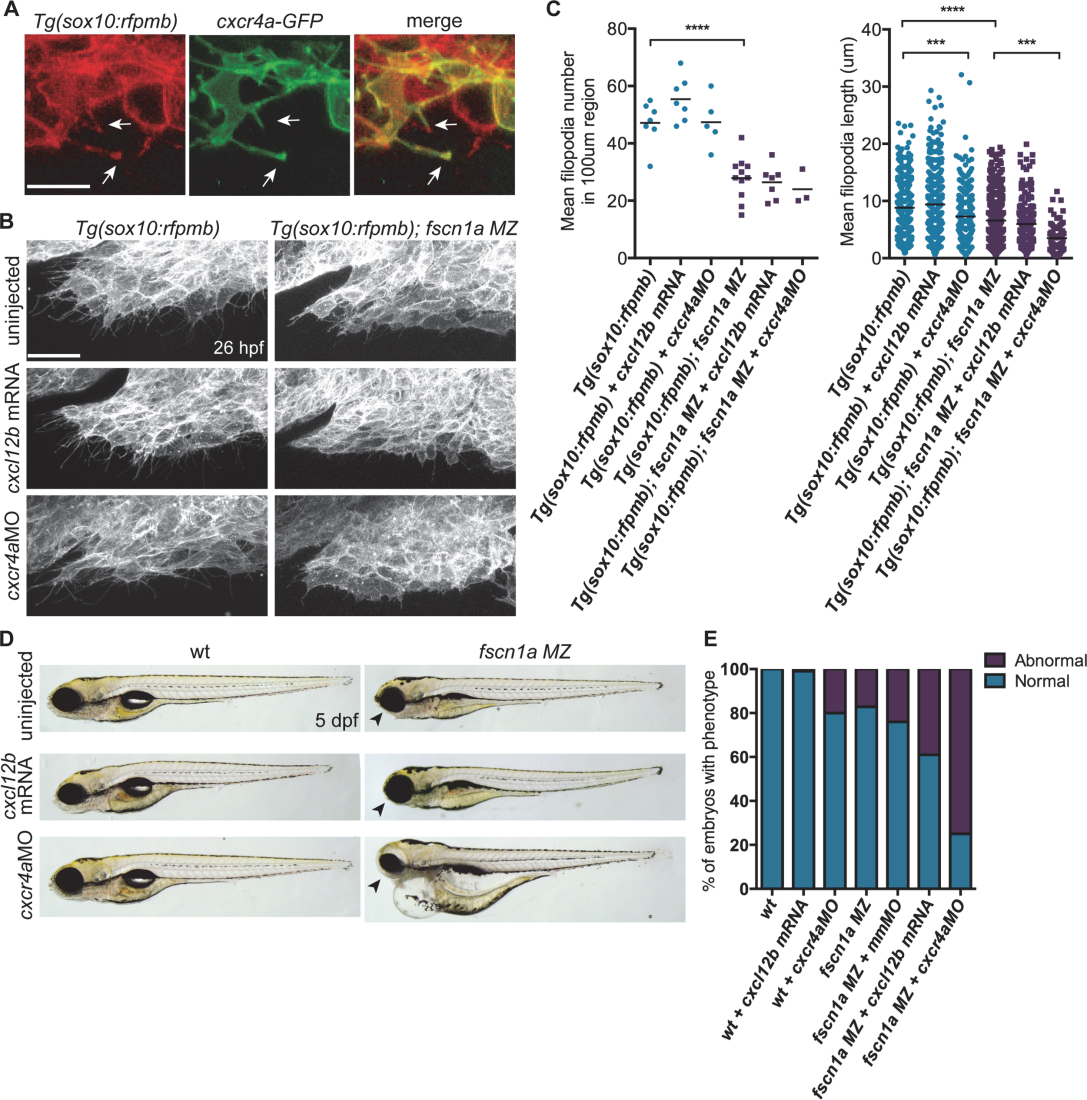
*fscn1a* cooperates with *cxcr4a/cxcl12b signaling* to regulate directional cranial NC migration. (A) Representative confocal images of cranial NC cells from a 26 hpf *Tg(sox10:rfpmb)* embryo injected with *cxcr4a-GFP* mRNA. Arrows highlight Cxcr4a-GFP localized to NC filopodia. Scale bar = 10 μm. (B) Lateral view of leading edge of cranial NC stream 1 in 26 hpf uninjected, *cxcl12b* mRNA-injected or *cxcr4aMO*-injected *Tg(sox10:rfpmb)* or *Tg(sox10:rfpmb); fscn1a MZ* embryos. Scale bar = 25 μm. (C) Quantitation of filopodia number and filopodia length in uninjected, *cxcl12b* mRNA-injected or *cxcr4aMO*-injected *Tg(sox10:rfpmb)* and *Tg(sox10:rfpmb); fscn1a MZ* embryos (n = 7 *Tg(sox10:rfpmb)*, n = 9 *Tg(sox10:rfpmb)* + *cxcl12b* mRNA, n = 5 *Tg(sox10:rfpmb)* + *cxcr4aMO*, n = 7 *Tg(sox10:rfpmb); fscn1a MZ*, n = 8 *Tg(sox10:rfpmb); fscn1a MZ* + *cxcl12b* mRNA, n = 3 *Tg(sox10:rfpmb); fscn1a MZ* + *cxcr4aMO*, ***p<0.001, ****p<0.0001). (D) Lateral views of 5 dpf uninjected, *cxcl12b* mRNA-injected or *cxcr4aMO*-injected wild type (wt) and *fscn1a MZ* embryos. Arrowheads indicate reduced jaw. (E) Quantitation of percentage of embryos with normal or abnormal craniofacial development at 5 dpf (n = 43 wt, 104 wt + *cxcl12b* mRNA, n = 51 wt + *cxcr4aMO*, n = 165 *fscn1a MZ*, n = 130 *fscn1a MZ* + *mmMO*, n = 123 *fscn1a MZ* + *cxcl12b* mRNA, n = 154 *fscn1a MZ* + *cxcr4aMO*).

Fscn1a is required for filopodia formation in all cranial NC streams, however defects in cell migration appear to be restricted to the first NC stream in *fscn1a MZ* embryos. This suggests that the few remaining filopodia in *fscn1a MZ* NC are sufficient to promote migration and/or additional cell migration mechanisms compensate for loss of chemoreception, adhesion, and/or motility in these cells. To identify cooperating mechanisms that regulate NC migration with *fscn1a*, we took advantage of the partially penetrant NC defects in *fscn1a MZ* embryos, as it represents a sensitized genetic background to identify interacting pathways. We took a candidate approach and focused on the chemokine receptor *cxcr4a* and its ligand *cxcl12b* (*sdf1b*). The *cxcr4/cxcl12* signaling axis regulates the directional migration of a variety of cell types [35,36] and has been previously demonstrated to regulate NC migration and development of NC-derived craniofacial cartilages and sympathetic ganglia [37,38]. Previous work in zebrafish demonstrated that *cxcr4a* is expressed in migratory cranial NC streams, while the *cxcl12b* ligand is expressed in pharyngeal endoderm, the target tissue of directionally migrating cranial NC cells [37]. Ectopic expression of *cxcr4a* or *cxcl12b* mRNA in zebrafish embryos uncouples *cxcr4a/cxcl12b*-dependent directional migration by randomizing the direction of the chemokine guidance signal to surrounding cells, thus causing cranial NC cells to migrate outside of their normal boundaries, resulting in loss of craniofacial cartilages [37]. Importantly, the craniofacial defects caused by *cxcr4a* or *cxcl12b* overexpression closely resemble the defects observed in *fscn1a MZ* embryos, including asymmetric loss of mandibular arch elements [37]. We first confirmed that Cxcr4a-GFP localizes to *fscn1a*-dependent filopodia in *Tg(sox10:rfpmb)* cranial NC cells (Fig. 8A). To determine if *fscn1a* cooperates with *cxcr4a/cxcl12b* to regulate directional NC migration, we ectopically expressed *cxcl12b* ligand in all surrounding cells or depleted *cxcr4a* receptor in NC cells by injecting wild type and *fscn1a* MZ embryos with subthreshold doses of *cxcl12b* mRNA or *cxcr4aMO*, respectively. NC cell filopodia were unaffected by misexpression of *cxcl12b* in 26 hpf *Tg(sox10:rfpmb)* and *Tg(sox10:rfpmb); fscn1a MZ* embryos, while low doses of *cxcr4aMO* reduced the length, but not number of filopodia in both *Tg(sox10:rfpmb)* and *Tg(sox10:rfpmb); fscn1a MZ* embryos (Fig. 8B,C). Low doses of *cxcl12b* mRNA or *cxcr4aMO* caused no defects in craniofacial cartilage formation (*cxcl12b* mRNA, 1/104) or mild defects in 20% (*cxcr4aMO*, 10/51) of wild type embryos (Fig. 8D-E). As previously observed, ∼20% (27/165) of *fscn1a MZ* embryos display defects in craniofacial development. Strikingly, the penetrance of this phenotype was increased to 40% (48/123) in *fscn1a MZ* embryos injected with *cxcl12b* mRNA or 75% (95/127) in *fscn1a MZ* embryos injected with *cxcr4aMO* (Fig. 8D-E, S9A Fig.). In addition, *cxcr4a* morphant/*fscn1a MZ* embryos displayed more severe morphological defects at 5 dpf (Fig. 8D). Importantly, injection of a control mismatch morpholino (*mmMO*) had no effect on the penetrance of craniofacial defects in *fscn1a MZ* embryos (Fig. 8E). Whole-mount ISH for *th* in 4 dpf embryos revealed that *fscn1a* and *cxcr4a* also function synergistically to promote development of the sympathetic ganglia (S9B-S9C Fig.). These genetic results suggest *fscn1a* functions in NC cells to mediate *cxcr4a/cxcl12b* chemokine signaling and may explain the partial penetrance of the *fscn1a MZ* NC phenotypes (see Discussion).

## Discussion

Coordinated migration of NC cells is essential for patterning the body plan of the vertebrate embryo. Multiple guidance mechanisms are employed by NC cells to ensure that they migrate to precise locations throughout the embryo, including cell-cell adhesion, chemo-attraction/repulsion and physical barriers [17,39,40]. A fundamental feature of the chemo-attraction/repulsion mechanism is the ability to sense the environment for guidance molecules, which is proposed to act through actin-rich filopodial protrusions [3,41]. In this study we generated zebrafish *fscn1a* null mutants to determine the relative contribution of *fscn1a*-dependent filopodia in patterning different NC migration pathways and formation of derivatives. Elimination of both zygotic and maternal *fscn1a* caused severe loss of filopodia in all migrating NC cells. Unexpectedly, we found that most NC cells migrate normally in *fscn1a* MZ mutants, suggesting that robust, long and numerous filopodia are not required for NC chemotaxis, at least in a subset of NC streams. Indeed, when wild type or *fscn1a* MZ mutants were treated with the F-actin depolymerizing drug Latrunculin B (Lat. B), which further depleted any residual filopodia, most NC cells still migrated normally. Instead, only a subset of collectively migrating NC cells show defects in directional cell migration in *fscn1a MZ* or *fscn1a MZ*/Lat. B-treated embryos: the NC cells that form the first pharyngeal arch and the peripheral sympathetic and enteric nervous systems. Disruption of the Cxcr4a/Cxcl12b signaling pathway significantly enhanced the severity of NC cell migration and derivative defects. Thus, Fscn1a functions with Cxcr4a/Cxcl12b chemokine signaling to promote directional migration of a subset of NC cells and subsequently pattern the craniofacial skeleton and sympathetic nervous system. These results also suggest that other mechanisms that regulate directional migration function redundantly with filopodia to ensure most NC cell populations reach their correct location.

### Perdurance of maternal protein rescues developmental functions of zygotic *fscn1a*

To determine the role of *fscn1a* in NC migration and development, we used TALEN-based gene targeting to generate multiple null alleles. Unexpectedly, we found that zygotic *fscn1a* is dispensable for development because maternal protein perdures throughout organogenesis for at least 10 days. In contrast, *fscn1a MZ* null mutant embryos display partially penetrant, but reproducible and heritable, defects in a subset of migrating NC cells and loss of a subset of NC-derived tissues. Thus, *fscn1a* mutants would have been missed in conventional forward genetic screens in zebrafish as well as maternal effect screens, which focus on mutants with early gastrula and embryonic phenotypes. As most genes required for NC development have been identified through forward genetic screens (or antisense morpholinos that do not affect oocyte-deposited proteins), we predict that additional genes required for NC development will be identified through similar TALENs and Cas9/CrispRs genome targeted approaches and subsequent examination of MZ mutants.

### Filopodia are required for directional migration in a subset of NC streams

As the antennae of the cell, the primary function of filopodia is presumed to be to sense the extracellular environment for directional cues and facilitate communication between migrating cells [3,41]. Live imaging studies *in vivo* have demonstrated that chick NC cells display dynamic short- and long-range filopodia during migration [42]. The functions of filopodia during chick NC migration is not known, but they have been proposed to initiate cell-cell adhesion and promote directional migration [42,43]. Our studies provide evidence that support a selective role for *fscn1a*-dependent filopodia in NC migration to promote directional migration of the whole stream into the first pharyngeal arch.

Unexpectedly, our results also demonstrate that most NC cells migrate normally in the absence of long, robustfilopodia. This may be due to redundant genetic pathways (see below), or to residual filopodia present in *fscn1a* mutant embryos. For example, fewer short-range filopodia may still localize enough cell-cell adhesion and receptor molecules to promote adhesion and directionality in posterior cranial NC streams and trunk NC. To test this, we used the actin depolymerizing drug Lat. B, which has been previously used in zebrafish to eliminate filopodia in endothelial cells during angiogenic sprouting [10]. Consistent with these findings, Lat. B treatment in wild type or *fscn1a MZ* mutants resulted in an almost complete absence of any filopodia-like protrusions, particularly in the *fscn1a MZ* mutants (Fig. 6), but did not cause any additional effects on NC migration or derivative formation. The few protrusions that remained were extremely short, wide, and flat and were not dynamic. Thus, while we cannot rule out the possibility that a single short membrane protrusion is sufficient for directional migration, we favor alternative models in which either localization of guidance receptors to the leading edge membrane is sufficient to promote directional migration and/or physical barriers, such as the budding endodermal pouches, overlying ectoderm and cranial placodes, provide a permissive environment for posterior NC streams to simply be pushed into the correct location without need for chemotaxis. Consistent with the latter model, disrupting endodermal pouch formation does not alter early NC migration but instead causes fusion of NC streams at later time points, which in turn selectively disrupts formation of the posterior branchial arches [34,44]. In addition, the first cranial NC stream migrates ∼4 hours before the endodermal pouches are formed and is the only stream affected by disrupting Cxcr4a/Cxcl12b signaling alone [37]. Thus, we propose that long, robust and dynamic filopodia are preferentially required in NC cells that migrate in the absence of surrounding physical barriers and/or are most dependent on long-range directional signaling through chemotaxis. This model may also explain the differential loss of sympathetic ganglia in *fscn1a MZ* mutants, as previous studies have shown that in vagal NC cells, Cxcr4 is required to sense Cxcl12 at the dorsal aorta to stop migration and aggregate to form ganglia [38].

### Redundant mechanisms of Fscn1 function during cell migration *in vivo*

Fscn1 belongs to a family of highly conserved actin bundling proteins required for filopodia formation [21]. Knockdown of *Fscn1* almost always decreases cell migration capacity *in vitro* [21,45,46]. In contrast, *fscn1* genetic mutants in *Drosophila* and mouse have tissue-specific phenotypes and are viable, suggesting *fscn1* is dispensable for embryogenesis *in vivo* [8,9,24–26,47]. The lack of *fscn1* embryonic phenotypes could be due to genetic redundancy with: 1) other actin-bundling proteins, such as villin, cofilin or espin, which have been shown to cooperate with *singed*/*fscn1* in cell culture and during *Drosophila* embryonic development [47–50], 2) parallel pathways that promote cell migration, as we demonstrate with Cxcr4a, or 3) maternal effects as discussed above. Indeed, in *Drosophila*, homozygous female *fscn1* mutants are sterile due to requirements for *fscn1* in ring canal formation during oogenesis, while the role of Fscn1 function in the mouse germline or early embryo remains unclear because it is not known if the *Fscn1* retroviral insertion affects *Fscn1* mRNA or protein levels in the oocyte or embryo before E14.5 [25]. Interestingly, mouse *Fscn1* mRNA is prominently expressed in the neural tube by E8.0 and pharyngeal arches by E9.5 [51] and approximately half of homozygous *Fscn1* mutant mouse pups die shortly after birth without milk in their stomach, suggesting a subtle or partially penetrant feeding or craniofacial defect [26].

Our studies show that *fscn1a MZ* loss results in ∼20% embryonic lethality, revealing an essential role for Fscn1 in embryogenesis. However, even in the absence of both maternal and zygotic *fscn1a*, most embryos develop to adulthood, suggesting other mechanisms compensate for *fscn1a* loss during embryogenesis. Indeed, by combining *fscn1a MZ* loss with *cxcl12b*-misexpression or partial *cxcr4a*-deficiency, we observed a dramatic increase in embryonic lethality, which at least in part, is due to defective NC development. Thus, our studies support a model in which *fscn1a* functions to promote directional migration of a subset of NC cells by localizing Cxcr4a to filopodia, which in turn allows efficient responses to local chemokine signals (such as Cxcl12b) in the first pharyngeal arch (mandibular cartilage) or adjacent to the dorsal aorta (sympathetic neurons); NC migration pathways previously shown to be selectively dependent on Cxcr4 signaling in fish and chick [37,38].

In recent years, Fscn1 has gained significant attention due to its upregulation in aggressive human carcinomas [22]. Assessment of FSCN1 as a potential target for anti-metastasis therapeutics is an area of active research [23,52–54]. Based on our results, we would predict that FSCN1 inhibitors would be relatively ineffective when used alone due to redundant mechanisms promoting cell migration and invasion. Instead, our studies support the idea that FSCN1 inhibitors be used in combination with other inhibitors of cell migration, particularly inhibitors that act on directional migration molecules like CXCR4. Future studies will be important to determine if Fscn1 functions redundantly with other actin cross-linking proteins, adhesion molecules and guidance cues in development and cancer invasion and metastasis, including NC-derived cancers like melanoma and neuroblastoma. Importantly, the *fscn1a MZ* mutants represent a sensitized genetic background to identify such cooperating mechanisms controlling cell migration in development and cancer.

## Methods

### Ethics statement

All experiments involving zebrafish conformed to the regulatory standards and guidelines of the University of Utah Institutional Animal Care and Use Committee (IACUC#12-11009). In accordance with IACUC standards, zebrafish embryos and adults were euthanized by immobilization by submersion in ice water (5 parts ice/1 part water) for at least 10 minutes following cessation of opercular movement.

### Animal husbandry and transgenic animals

Zebrafish were maintained and bred as described [55]. Transgenic lines used were described previously: *Tg(sox10:RFPmb)* [32], *Tg(sox10:GFP)* [56], *Tg(ngn1:gfp)* [57], *Tg(phox2b:gfp)* [58] and *Tg(dbh:gfp)* [59].

### Molecular biology and cloning

Based on published GenBank sequences, the coding sequences of zebrafish *fscn1a* (Gene ID: 558271), *fscn1b* (Gene ID: 570314), *fscn2a* (Gene ID: 798075), and *fscn2b* (Gene ID: 393743) were PCR amplified from a 24 hpf (*fscn1a*) or 48 hpf (*fscn1b, fscn2a, fscn2b*) zebrafish cDNA library and TOPO cloned into pGEM-T Easy (Promega) or directionally cloned into pCS2.

### Generation and genotyping of *fscn1a* mutant

A pair of TALEN plasmids targeting zebrafish *fscn1a* was designed and constructed by the University of Utah Mutation Generation and Detection Core (http://www.cores.utah.edu/) as previously described [60,61]. TALEN target sites were designed using the TALEN Effector Nucleotide Targeter 2.0 program [62] at https://tale-nt.cac.cornell.edu. The TALEN Golden Gate kit [63] was used with modifications to construct the *fscn1a* TALEN plasmids. Plasmids were linearized with Not1 and *in vitro* transcription was carried out using SP6 mMESSAGE mMACHINE kit (Ambion). 50 pg of each mRNA was microinjected into the yolk of 100 one-cell AB embryos.

To identify *fscn1a* mutant adults or embryos, genomic DNA was isolated from fin clips or tail clips, respectively. A 111 bp amplicon spanning the mutation site was isolated by PCR using LightScanner Master Mix (Biofire) and analyzed by high resolution melt analysis (HRMA) on a LightScanner (Biofire) as previously described [60,64]. To distinguish homozygous *fscn1a* mutants from wild type, all unidentified samples that grouped with known wild type samples were spiked with 2 μl wild type reaction, heated to 95° for 5 minutes, and re-analyzed on the LightScanner. The melt curve for homozygous *fscn1a* mutant samples shifted to a heterozygous *fscn1a* mutant melt curve, while the melt curve for wild type samples did not change. Wild type, heterozygous and homozygous *fscn1a* melt curves were validated by sequencing.

### Preparation of embryonic protein lysates

After tail clips were taken, each embryo of unknown genotype was placed in 8 μl cold modified RIPA buffer with PI cocktail (1:100, Roche) on ice. Samples were briefly homogenized using a sterile pipette tip and stored at −80° until genotyping was complete. Samples of the same genotype were pooled and protein concentration was determined by BCA Protein Assay (Pierce). Protein samples were diluted to 0.4 μg/μL in 1X LDS (prepared from 4X LDS, Invitrogen). To prepare lysates from non-genotyped embryos, 10 embryos were added to 80 μl modified RIPA buffer PI cocktail and homogenized using a sterile pestle.

### Alcian blue staining, whole-mount in situ hybridization and immunostaining

Cartilage staining with Alcian blue and whole-mount in situ hybridization was carried out as previously described [65,66]. Antisense RNA probes against zebrafish *fscn1a* or *fscn1b* were generated by digestion of pGEM-*fscn1a* with Kpn1 or Nco1 and subsequent *in vitro* transcription with T7 or SP6 RNA polymerase, respectively. Antisense RNA probes were generated for *foxd3, crestin, dlx2a, sox10, th*, and *nkx2.3* as described [27,67]. Whole-mount immunostaining for GFP was performed on embryos fixed in 4% PFA overnight at 4° and permeabilized in methanol for 2 hours at −20°. Antibodies used in this study include: chicken anti-GFP (1:1000, Aves), goat anti-chicken 488 (1:250, Invitrogen), rabbit anti-FSCN1 (1:2000, Sigma) and mouse anti-GAPDH (1:1000, Abcam).

### Morpholino and mRNA microinjection

Previously published antisense morpholino oligonucleotides against *foxd3, tfap2a*, and *cxcr4a* were used [27,37,68]. To generate full-length capped sense mRNA, pCS2-*fscn1a*, pCS2-*cxcr4aGFP*, or pCS2-*cxcl12b* was linearized with Not1 and *in vitro* transcription was carried out using SP6 mMESSAGE mMACHINE kit (Ambion). Morpholinos or mRNA were microinjected into the yolk of one-cell embryos at the following dosages: 1.25 ng *foxd3MO*, 1.25 ng *tfap2aMO*, 1.25 ng *fscn1bMO*, 1.25 ng *fscn2aMO*, 1.25 ng *fscn2bMO*, 1.5 ng *cxcr4aMO*, 25 pg *fscn1a* mRNA, 25 pg *cxcr4a-GFP* mRNA, 7.5 pg *cxcl12b* mRNA.

### Latrunculin B treatment

Latrunculin B (Lat. B, Sigma-Aldrich Cat. # L5288) was reconstituted to 15 mg/mL in DMSO. A working stock was prepared fresh for each experiment by diluting Lat. B to 5 μg/ml in egg water (1:3000). A DMSO working stock was prepared by diluting DMSO in egg water (1:3000). To treat embryos, 10–15 dechorionated 10 hpf embryos were placed in 1 well of a 12 well plate with 2 mL egg water and working stocks of Lat. B or DMSO were diluted to the final experimental concentration. Guided by previous studies with Lat. B-treated zebrafish embryos [10], dose response curves were performed to determine an optimal dose of Lat. B that inhibited filopodia formation but did not affect other actin structures or cause lethality. Initial dose response experiments were performed with 25 ng/ml (10 μl working stock), 50 ng/ml (20 μl), 75 ng/ml (30 μl), 100 ng/ml (40 μl) and 150 ng/ml (60 μl) Lat. B or DMSO. A second dose response was performed with 75 ng/ml (30 μl working stock), 85 ng/ml (34 μl) and 95 ng/ml (38 μl) Lat. B or DMSO. From these dose response experiments, 80 ng/ml (32 μl) Lat. B or DMSO was determined to be the optimal dose of Lat. B for inhibiting filopodia formation in NC cells. 80 ng/ml Lat. B was used in all subsequent experiments. Selective inhibition of F-actin in residual NC cell filopodia was validated by monitoring F-actin polymerization using *lifeact-GFP* mRNA injected into *Tg(sox10:rfpmb)* embryos with low dose Lat. B or DMSO and visualizing filopodia by fluorescent confocal microscopy (S10 Fig.).

### Image acquisition and processing

Confocal images were acquired using an Olympus Fluoview FV1200 confocal microscope and Olympus FV10-ASW v4.1 software. Olympus UPlanSApo 60X/1.20W and Olympus UPlanSApo 10X/0.45 objectives were used in this study. For all confocal imaging, embryos were embedded on cover slips in 1% low melt agarose. For analysis of filopodia dynamics, z-stacks of the leading edge of NC streams 1–2 (cranial, n = 6) or of trunk NC cells between somites 6–8 (trunk, n = 6) in 26 hpf *Tg(sox10:rfpmb)* and *Tg(sox10:rfpmb); fscn1a MZ embryos* were acquired every 4 minutes for one hour using the 60X water objective (S1–S2 Movies). To monitor NC migration and individual cell behaviors, z-stacks were acquired every 25 minutes for 18 hours using the 10X objective (S3–S4 Movies). Widefield fluorescent images were acquired on an Olympus SZX16 microscope configured with an Olympus DP72 camera. Brightfield images were taken using a Nikon C-DSD115 microscope configured with an Olympus DP72 camera. Prism 6, ImageJ 1.46r, Adobe Photoshop CS5 and CS6, and Adobe Illustrator CS6 were used to generate figures.

### Quantification and statistical analysis

**Filopodia characterization in wild type and fscn1a MZ embryos**. Filopodia number and length was measured using ImageJ 1.46r. Filopodia within a 20 μm region at the leading edge of NC stream 2 were included in analyses. For each experimental condition, 5 filopodial protrusions from 6 embryos were analyzed (n = 30). Filopodia from *Tg(sox10:rfpmb)* and *Tg(sox10:rfpmb); fscn1a MZ* embryos were compared using an unpaired t-test (Prism 6). Significance is denoted with asterisks. ****p<0.0001.

**Filopodia number and length in embryos injected with cxcl12b mRNA, cxcr4aMO, or treated with Latrunculin B**. Filopodia number and length was measured using ImageJ 1.46r. All filopodia within a 100 μm region at the leading edge of NC stream 1 were included in analyses. Filopodia number and length were compared using an unpaired t-test (Prism 6). Significance is denoted with asterisks. *p<0.05, **p<0.005, ***p<0.001, ****p<0.0001.

**Sympathetic ganglia**. To detect sympathetic ganglia, ISH for *th* was performed on 3 dpf wt (n = 25) or *fscn1a MZ* (n = 24) embryos. In ImageJ, all RGB images were split into separate channels and the red channel was used for analysis. Color Threshold was adjusted using the Default method to 0–150 and the Analyze Particles feature was used to measure the area (μm^2^) of *th+* sympathetic ganglia. Area of sympathetic ganglia was compared using an unpaired t-test (Prism 6). Significance is denoted with asterisks. *p<0.05, **p<0.005, ***p<0.001, ****p<0.0001, ns = not significant.

**Enhanced penetrance of fscn1a MZ craniofacial defects**. Fisher’s exact test (Prism 6) was used to determine if treatment with Latrunculin B or injection of *cxcl12b* mRNA or *cxcr4aMO* enhanced the penetrance of craniofacial defects in *fscn1a MZ* embryos. Significance is denoted with asterisks. *p<0.05.

**Enteric neurons**. *Tg(phox2b:gfp)* (n = 18) and *Tg(phox2b:gfp); fscn1aMZ* (n = 15) 3 dpf embryos were imaged and used for analysis. *phox2b+* enteric neurons within a 500 pixel (0.625μm) × 100 pixel (0.125μm) region were counted in ImageJ. To enable automated counting, all images were processed to Find Edges and Color Threshold was adjusted using the Yen method. The Analyze Particles feature was used to count all particles with a circularity between 0.1 and 1.0. Number of enteric neurons was compared using an unpaired t-test (Prism 6). Significance is denoted with asterisks. *p<0.05.

## Supporting Information

S1 Fig Expression of *fscn1b* during embryonic development. (A) Whole-mount ISH for *fscn1b* mRNA expression at 11 hpf (3 somite), 18 hpf (18 somite), 24 hpf, 36 hpf, and 48 hpf embryos. *fscn1b* expression is observed in specific neurons in the developing brain at 36 hpf and 48 hpf (arrows). (B) Whole-mount ISH for *fscn1b* mRNA expression in 36 hpf embryos using a control sense RNA probe. Black pigment in eyes, trunk and over yolk is visible.
(TIF)

S2 Fig Expression of *fscn1a* in trunk NC. (A) Lateral views of *fscn1a* or *crestin* mRNA expression in 18 hpf embryos. Trunk is enlarged in lower panels. Arrows highlight NC expression. Scale bar = 100 μm. (B) Lateral views of *fscn1a* mRNA expression in a 24 hpf embryo (top panel) and with *crestin* (bottom panels) showing co-expression in NC but not vasculogenic mesoderm (vm). Boxed region is enlarged in right panel. Scale bar = 100 μm. (C) Whole-mount ISH for *fscn1a* mRNA expression in 24 hpf embryos using a control sense RNA probe.
(TIF)

S3 Fig Generation of *fscn1a* null alleles by TALEN-induced mutagenesis. (A) Schematic of the *fscn1a* genomic locus. TALEN binding sites in exon1 are depicted. Arrow indicates translation start site. (B) Outline of breeding strategy to isolate *fscn1a* mutant alleles and generate *fscn1a* zygotic (*zyg*) and maternal-zygotic (*MZ)* mutant zebrafish. The *fscn1a* TALENs were injected into one-cell stage embryos and G0 mosaic adults analyzed for new mutations. G0 adults were outcrossed to wild-type animals to generate multiple independent families with unique *fscn1a* mutations. Heterozygous *fscn1a* adults from each family were incrossed to generate 25% zygotic homozygous embryos, which were viable and fertile. Zygotic homozygous adults were then incrossed to generate 100% *fscn1a MZ* embryos. (C) Nucleotide sequences of *fscn1a* mutant alleles showing locations of different deletions and insertions that create nonsense mutations (red). (D) Schematic of Fscn1a protein structure, including N-terminal and C-terminal actin binding sites and S39 regulatory residue.
(TIF)

S4 Fig *fscn1a* mRNA and Fscn1a protein are expressed in unfertilized wild type oocytes. (A) Relative expression of *fscn1a* visualized on Integrated Genome Browser (IGB) for egg, one cell, 16–32 cell, 128–258 cell, 3.5hpf and 5.3hpf [69] aligned to the zv9 genome. Scale: 0–10 for egg, one cell, 16–32 cell, 128–258 cell, 3.5hpf, and scale: 0–40 for 5.3hpf. (B) Western blot showing Fscn1a protein levels in wild type (wt) unfertilized eggs, as well as wild type, zygotic *fscn1a* mutant (*fscn1a zyg*), and maternal-zygotic *fscn1a* mutant (*fscn1a MZ*) embryos at 24 hpf. Numbers on right side indicate molecular mass markers.
(TIF)

S5 Fig *fscn* paralogs are not aberrantly expressed in *fscn1a MZ* mutant embryos. Expression of *fscn1a, fscn1b, fscn2a, fscn2b* and *gapdh* in wild type and *fscn1a MZ* embryos at 11 hpf, 14 hpf, 18 hpf and 26 hpf was determined by RT-PCR. No aberrant expression (e.g. upregulation) of zebrafish *fscn* paralogs was observed in *fscn1a MZ* embryos.
(TIF)

S6 Fig Loss of *fscn* paralogs does not enhance *fscn1a MZ* craniofacial defects. Ventral views of 5 dpf uninjected or *fscn1b/2a/2bMO*-injected wild type and *fscn1a MZ* embryos stained with Alcian blue. Numbers in parentheses indicate number of embryos with depicted phenotype.
(TIF)

S7 Fig *fscn1a MZ* embryos display abnormal craniofacial skeleton morphology. Representative images of craniofacial skeleton phenotypes observed in *fscn1a MZ* embryos. Ventral views of craniofacial skeleton in 5 dpf *Tg(sox10:rfpmb); fscn1a MZ* embryos. Phenotypes are grouped into three classes: normal craniofacial cartilage morphology (Class I), mild deformation of mandibular arch (Class II) and loss of mandibular arch elements (Class III). Within a clutch of *fscn1a MZ* embryos, ∼80% of embryos belong to Class I or II. The remaining 20% of embryos display symmetric or asymmetric loss of mandibular arch elements of variable severity and belong to Class III. Numbers denote pharyngeal arches, ma = mandibular arch (arch 1), ep = ethmoid plate. Scale bar = 100 μm.
(TIF)

S8 Fig *nkx2.3+* pharyngeal endoderm is patterned normally in *fscn1a MZ* embryos. Whole-mount in situ hybridyzation for *nkx2.3* in wild type and *fscn1a MZ* embryos at 30 hpf showing normal endodermal (end) pouch formation.
(TIF)

S9 Fig *fscn1a* cooperates with *cxcr4a/cxcl12b signaling* to regulate development of a subset of NC derivatives. (A) Representative ventral views of 5 dpf uninjected or *cxcl12b* mRNA-injected wild type and *fscn1a MZ* embryos stained with Alcian blue. (B) Whole-mount ISH for *th* expression in 4 dpf uninjected or *cxcr4aMO*-injected wt and *fscn1a MZ* embryos. (C) Average area of *th+* sympathetic ganglia (n = 8 wt, 15 wt + *cxcr4aMO*, 17 *fscn1a MZ*, 17 *fscn1a MZ* + *cxcr4aMO*, **p = 0.0039, ***p = 0.0003, ****p<0.0001, sym = sympathetic ganglia).
(TIF)

S10 Fig Low dose Latrunculin B treatment inhibits F-actin polymerization in residual NC cell filopodia. Representative maximum projection confocal images of the leading edge of second cranial NC stream from 26 hpf *Tg(sox10:rfpmb)* embryos injected with *lifeact-GFP* mRNA and treated with low dose Lat. B or DMSO. Arrows indicate filopodia. Asterisk denotes lack of Lifeact-GFP in RFP+ filopodia in Lat. B-treated embryo. F-actin in underlying yolk cells is also visible in all images.
(TIF)

S1 Movie Lateral view of NC stream 2 in 26 hpf *Tg(sox10:rfpmb)* embryo. Note extensive filopodia dynamics at leading edge of stream.
(AVI)

S2 Movie Lateral view of NC stream 2 in 26 hpf *Tg(sox10:rfpmb); fscn1a MZ* embryo. Filopodia at leading edge are reduced in number and dynamics.
(AVI)

S3 Movie Lateral view of NC streams 1 and 2 in *Tg(sox10:rfpmb)* embryo from ∼16 hpf – 31 hpf. (AVI)

S4 Movie Lateral view of NC streams 1 and 2 in *Tg(sox10:rfpmb); fscn1a MZ* embryo from ∼16 hpf – 31 hpf. (AVI)
