# Supplementary figures and images for: Fascin1-Dependent Filopodia are Required for Directional Migration of a Subset of Neural Crest Cells
Source: PLoS Genet. 2015 Jan 21;11(1):e1004946. doi: 10.1371/journal.pgen.1004946 (PMC4301650; doi:10.1371/journal.pgen.1004946)

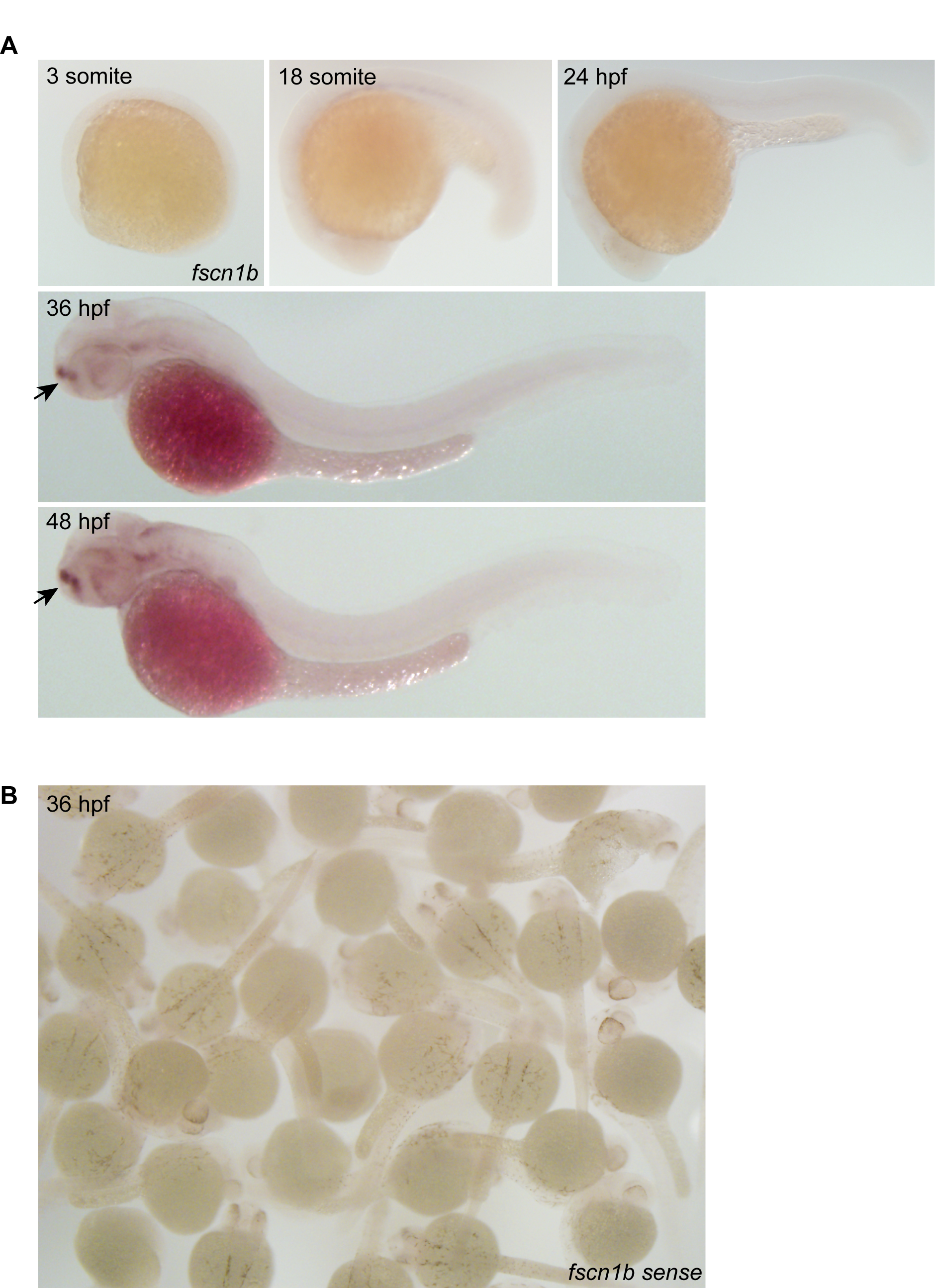

Supplement: S1 Fig — (A) Whole-mount ISH for fscn1b mRNA expression at 11 hpf (3 somite), 18 hpf (18 somite), 24 hpf, 36 hpf, and 48 hpf embryos. fscn1b expression is observed in specific neurons in the developing brain at 36 hpf and 48 hpf (arrows). (B) Whole-mount ISH for fscn1b mRNA expression in 36 hpf embryos using a control sense RNA probe. Black pigment in eyes, trunk and over yolk is visible. (TIF) [file pgen.1004946.s001.tif]

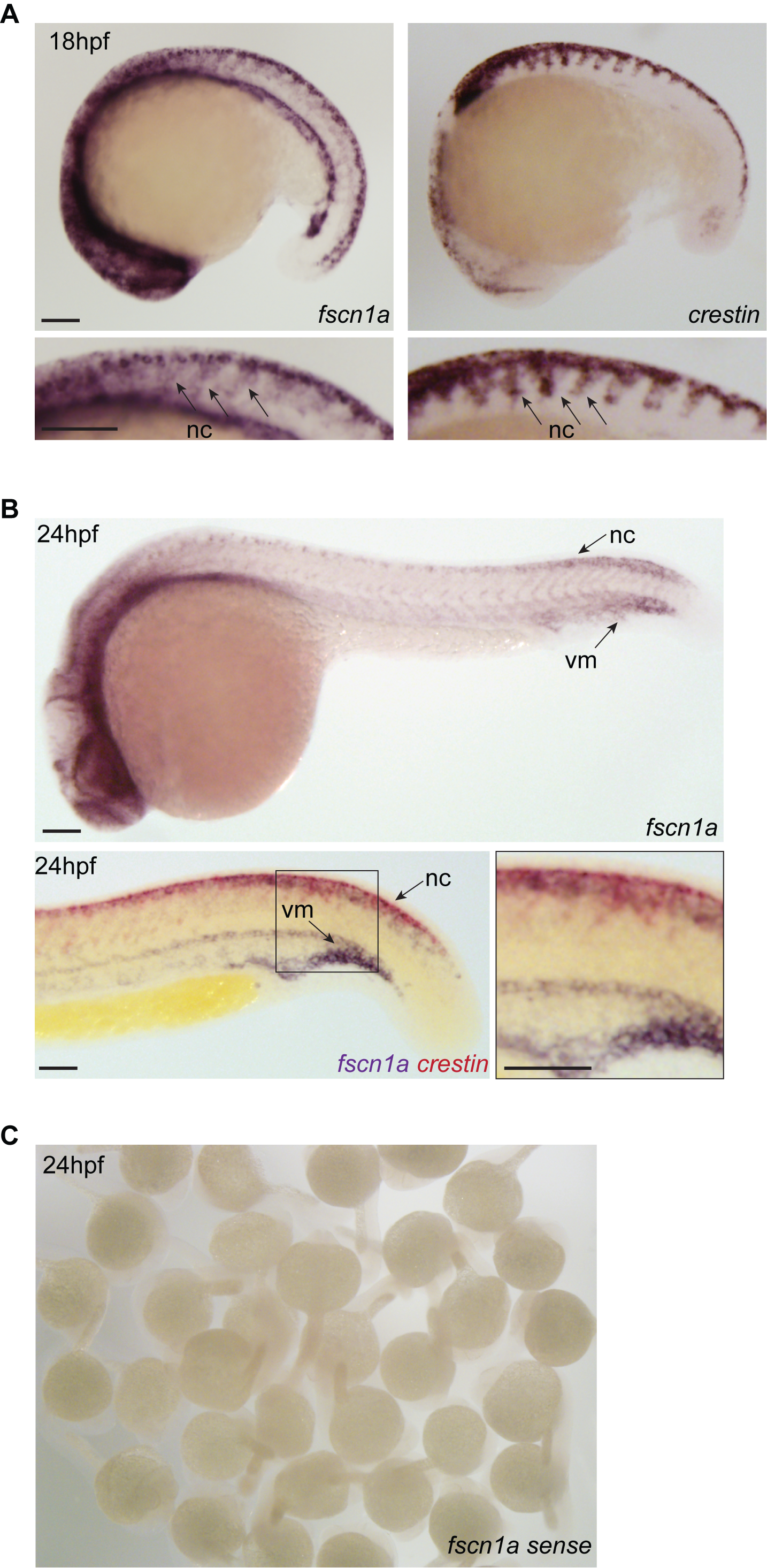

Supplement: S2 Fig — (A) Lateral views of fscn1a or crestin mRNA expression in 18 hpf embryos. Trunk is enlarged in lower panels. Arrows highlight NC expression. Scale bar = 100 μm. (B) Lateral views of fscn1a mRNA expression in a 24 hpf embryo (top panel) and with crestin (bottom panels) showing co-expression in NC but not vasculogenic mesoderm (vm). Boxed region is enlarged in right panel. Scale bar = 100 μm. (C) Whole-mount ISH for fscn1a mRNA expression in 24 hpf embryos using a control sense RNA probe. (TIF) [file pgen.1004946.s002.tif]

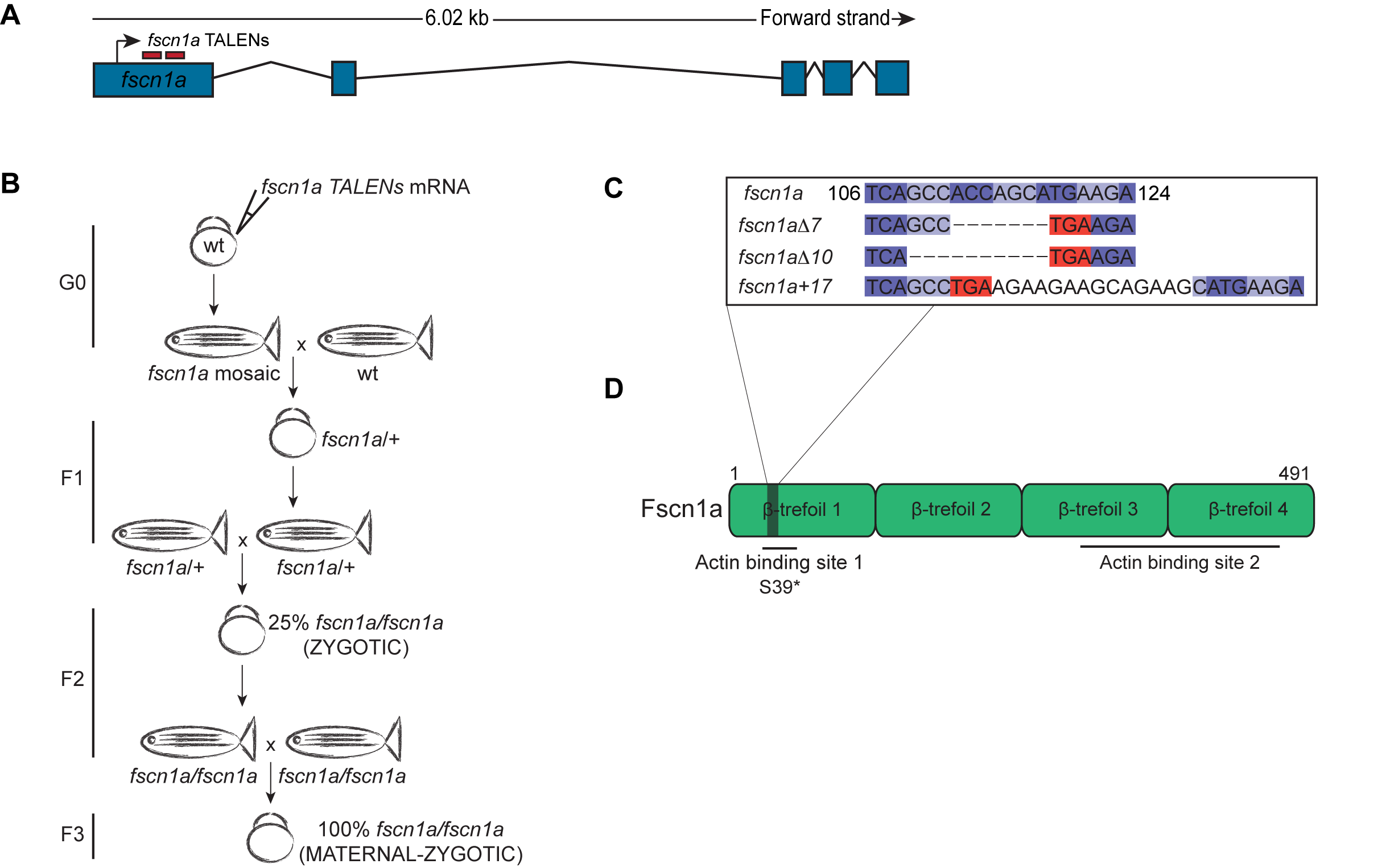

Supplement: S3 Fig — (A) Schematic of the fscn1a genomic locus. TALEN binding sites in exon1 are depicted. Arrow indicates translation start site. (B) Outline of breeding strategy to isolate fscn1a mutant alleles and generate fscn1a zygotic (zyg) and maternal-zygotic (MZ) mutant zebrafish. The fscn1a TALENs were injected into one-cell stage embryos and G0 mosaic adults analyzed for new mutations. G0 adults were outcrossed to wild-type animals to generate multiple independent families with unique fscn1a mutations. Heterozygous fscn1a adults from each family were incrossed to generate 25% zygotic homozygous embryos, which were viable and fertile. Zygotic homozygous adults were then incrossed to generate 100% fscn1a MZ embryos. (C) Nucleotide sequences of fscn1a mutant alleles showing locations of different deletions and insertions that create nonsense mutations (red). (D) Schematic of Fscn1a protein structure, including N-terminal and C-terminal actin binding sites and S39 regulatory residue. (TIF) [file pgen.1004946.s003.tif]

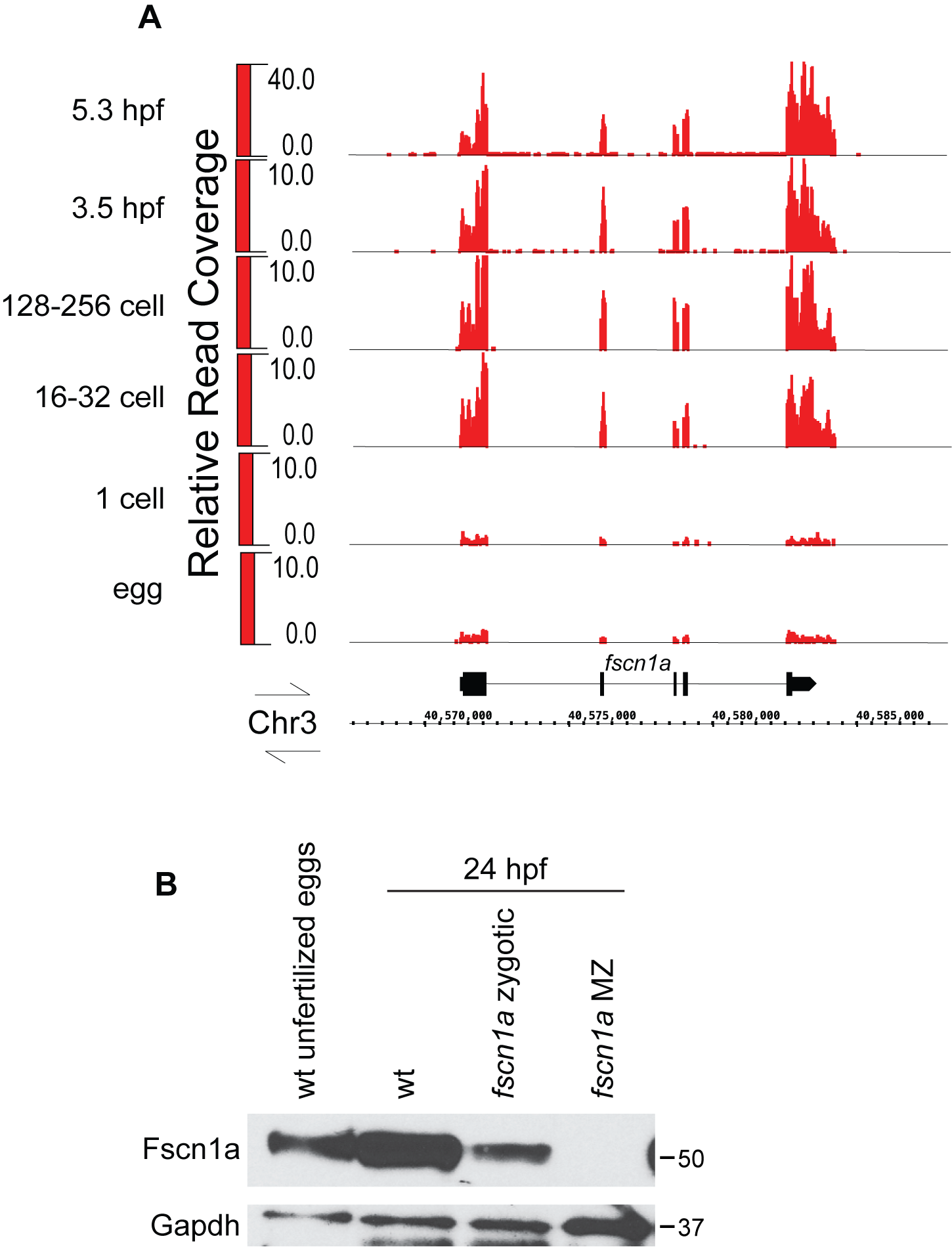

Supplement: S4 Fig — (A) Relative expression of fscn1a visualized on Integrated Genome Browser (IGB) for egg, one cell, 16–32 cell, 128–258 cell, 3.5hpf and 5.3hpf [69] aligned to the zv9 genome. Scale: 0–10 for egg, one cell, 16–32 cell, 128–258 cell, 3.5hpf, and scale: 0–40 for 5.3hpf. (B) Western blot showing Fscn1a protein levels in wild type (wt) unfertilized eggs, as well as wild type, zygotic fscn1a mutant (fscn1a zyg), and maternal-zygotic fscn1a mutant (fscn1a MZ) embryos at 24 hpf. Numbers on right side indicate molecular mass markers. (TIF) [file pgen.1004946.s004.tif]

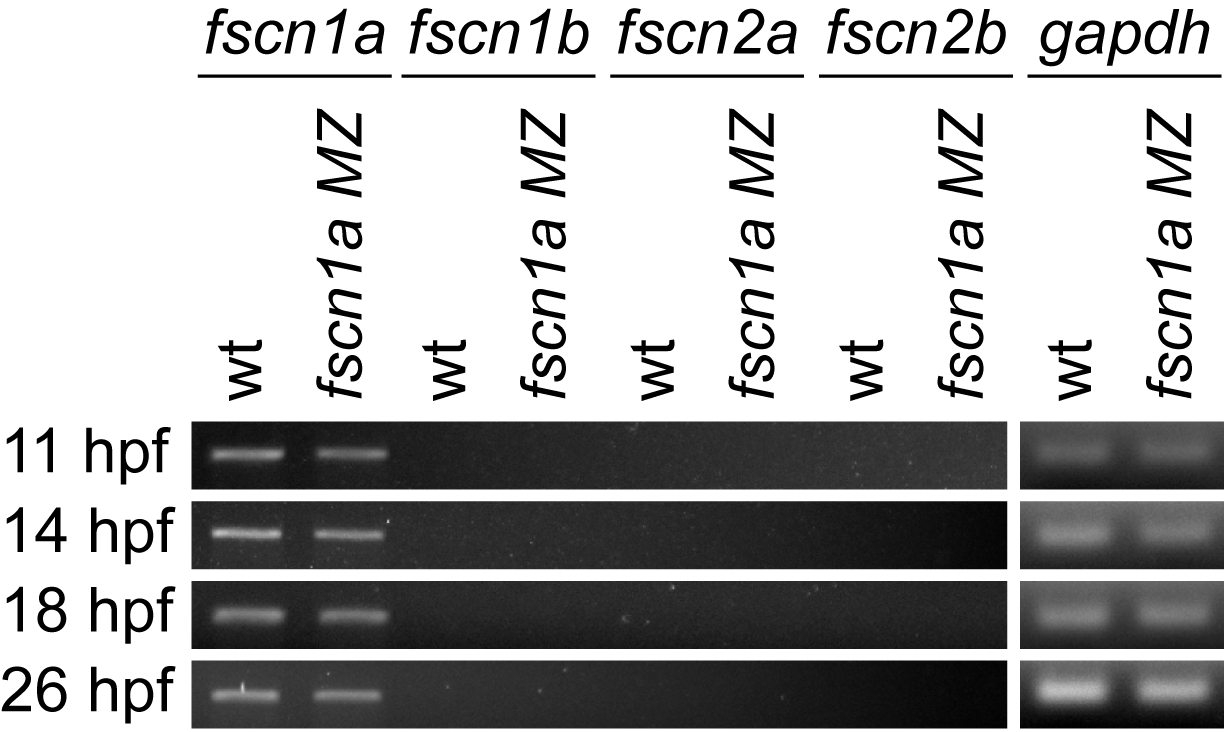

Supplement: S5 Fig — Expression of fscn1a, fscn1b, fscn2a, fscn2b and gapdh in wild type and fscn1a MZ embryos at 11 hpf, 14 hpf, 18 hpf and 26 hpf was determined by RT-PCR. No aberrant expression (e.g. upregulation) of zebrafish fscn paralogs was observed in fscn1a MZ embryos. (TIF) [file pgen.1004946.s005.tif]

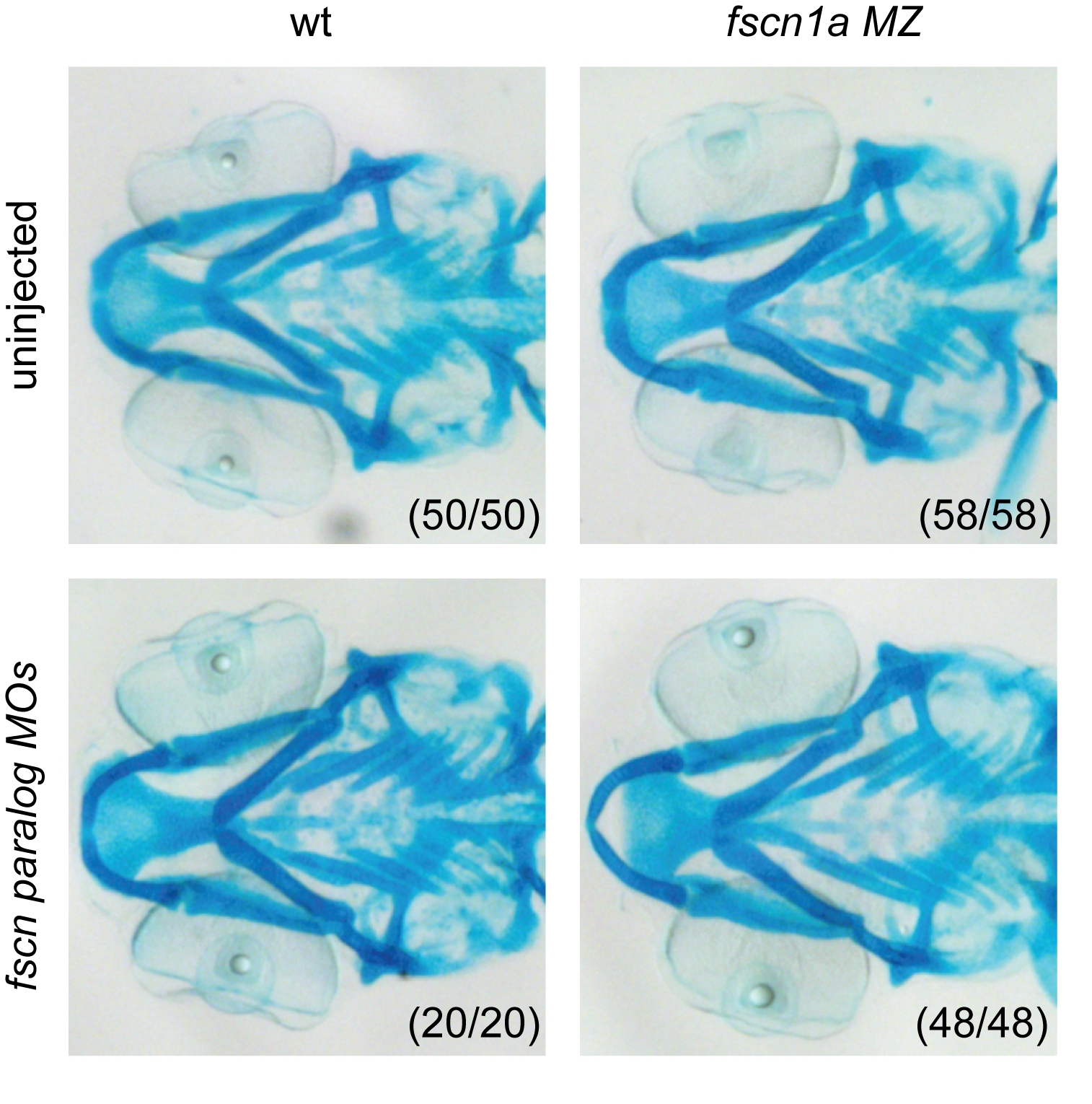

Supplement: S6 Fig — Ventral views of 5 dpf uninjected or fscn1b/2a/2bMO-injected wild type and fscn1a MZ embryos stained with Alcian blue. Numbers in parentheses indicate number of embryos with depicted phenotype. (TIF) [file pgen.1004946.s006.tif]

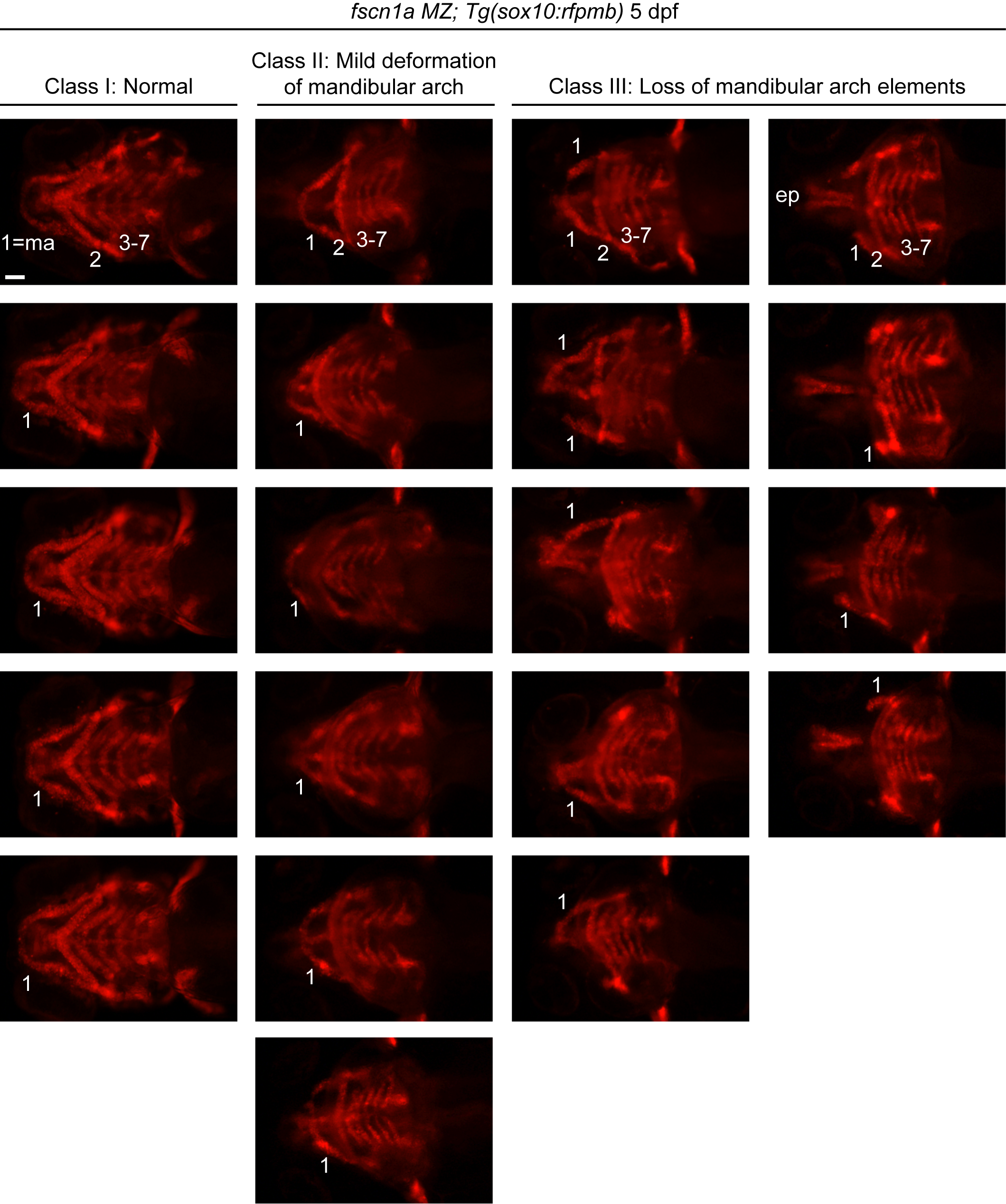

Supplement: S7 Fig — Representative images of craniofacial skeleton phenotypes observed in fscn1a MZ embryos. Ventral views of craniofacial skeleton in 5 dpf Tg(sox10:rfpmb); fscn1a MZ embryos. Phenotypes are grouped into three classes: normal craniofacial cartilage morphology (Class I), mild deformation of mandibular arch (Class II) and loss of mandibular arch elements (Class III). Within a clutch of fscn1a MZ embryos, ∼80% of embryos belong to Class I or II. The remaining 20% of embryos display symmetric or asymmetric loss of mandibular arch elements of variable severity and belong to Class III. Numbers denote pharyngeal arches, ma = mandibular arch (arch 1), ep = ethmoid plate. Scale bar = 100 μm. (TIF) [file pgen.1004946.s007.tif]

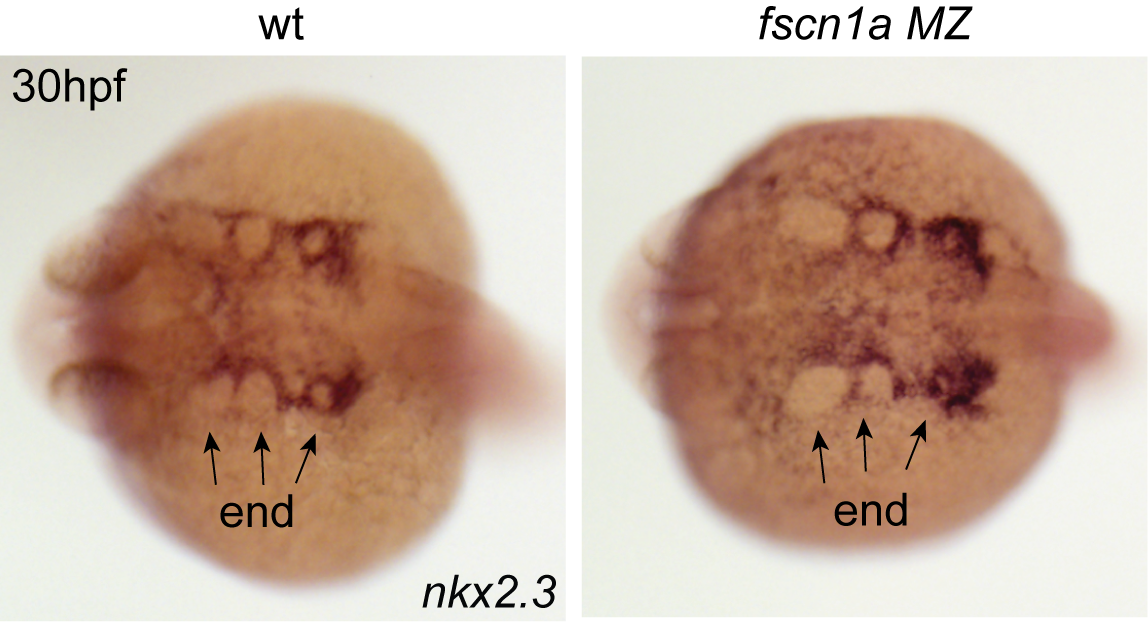

Supplement: S8 Fig — Whole-mount in situ hybridyzation for nkx2.3 in wild type and fscn1a MZ embryos at 30 hpf showing normal endodermal (end) pouch formation. (TIF) [file pgen.1004946.s008.tif]

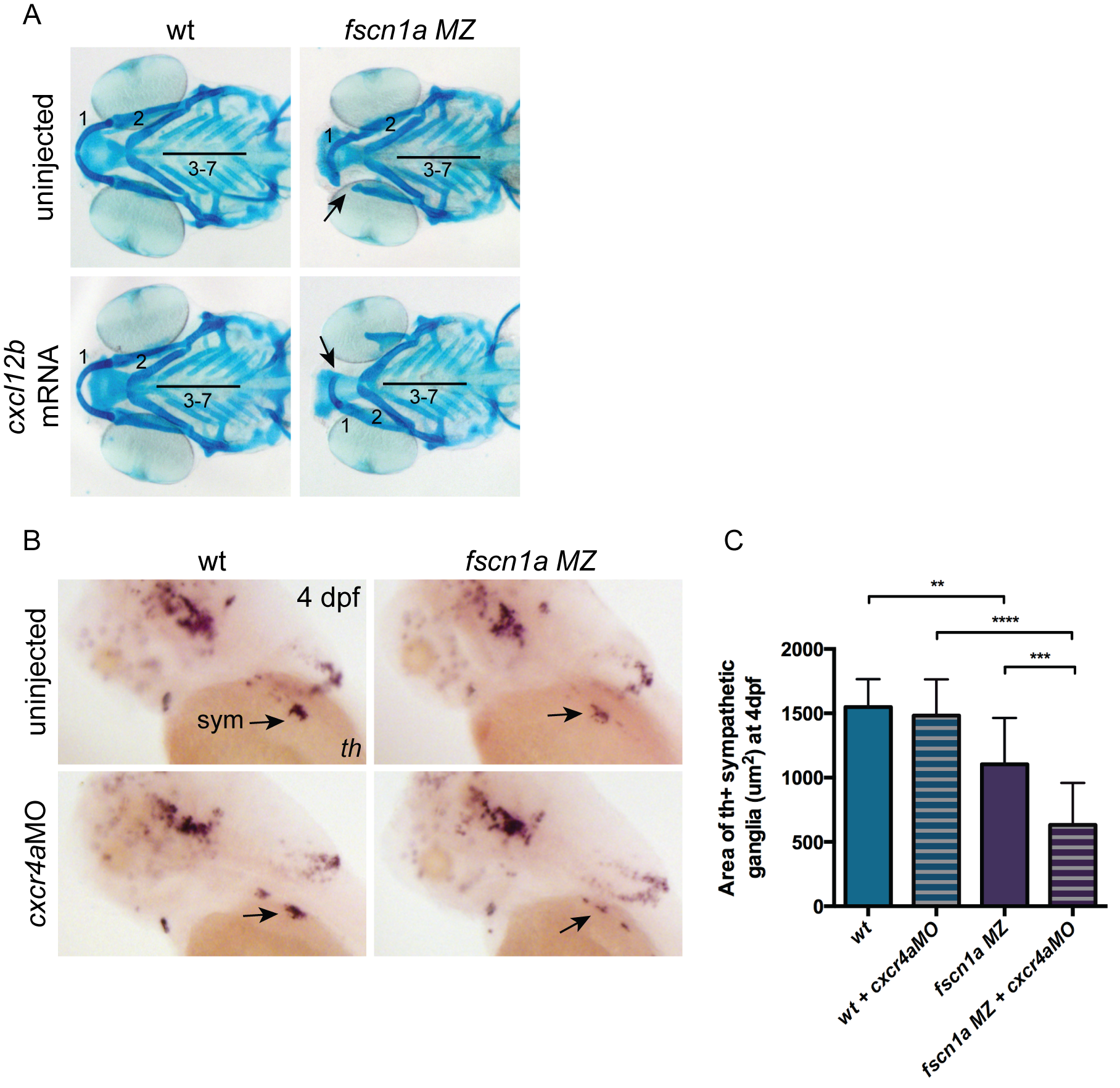

Supplement: S9 Fig — (A) Representative ventral views of 5 dpf uninjected or cxcl12b mRNA-injected wild type and fscn1a MZ embryos stained with Alcian blue. (B) Whole-mount ISH for th expression in 4 dpf uninjected or cxcr4aMO-injected wt and fscn1a MZ embryos. (C) Average area of th+ sympathetic ganglia (n = 8 wt, 15 wt + cxcr4aMO, 17 fscn1a MZ, 17 fscn1a MZ + cxcr4aMO, **p = 0.0039, ***p = 0.0003, ****p<0.0001, sym = sympathetic ganglia). (TIF) [file pgen.1004946.s009.tif]

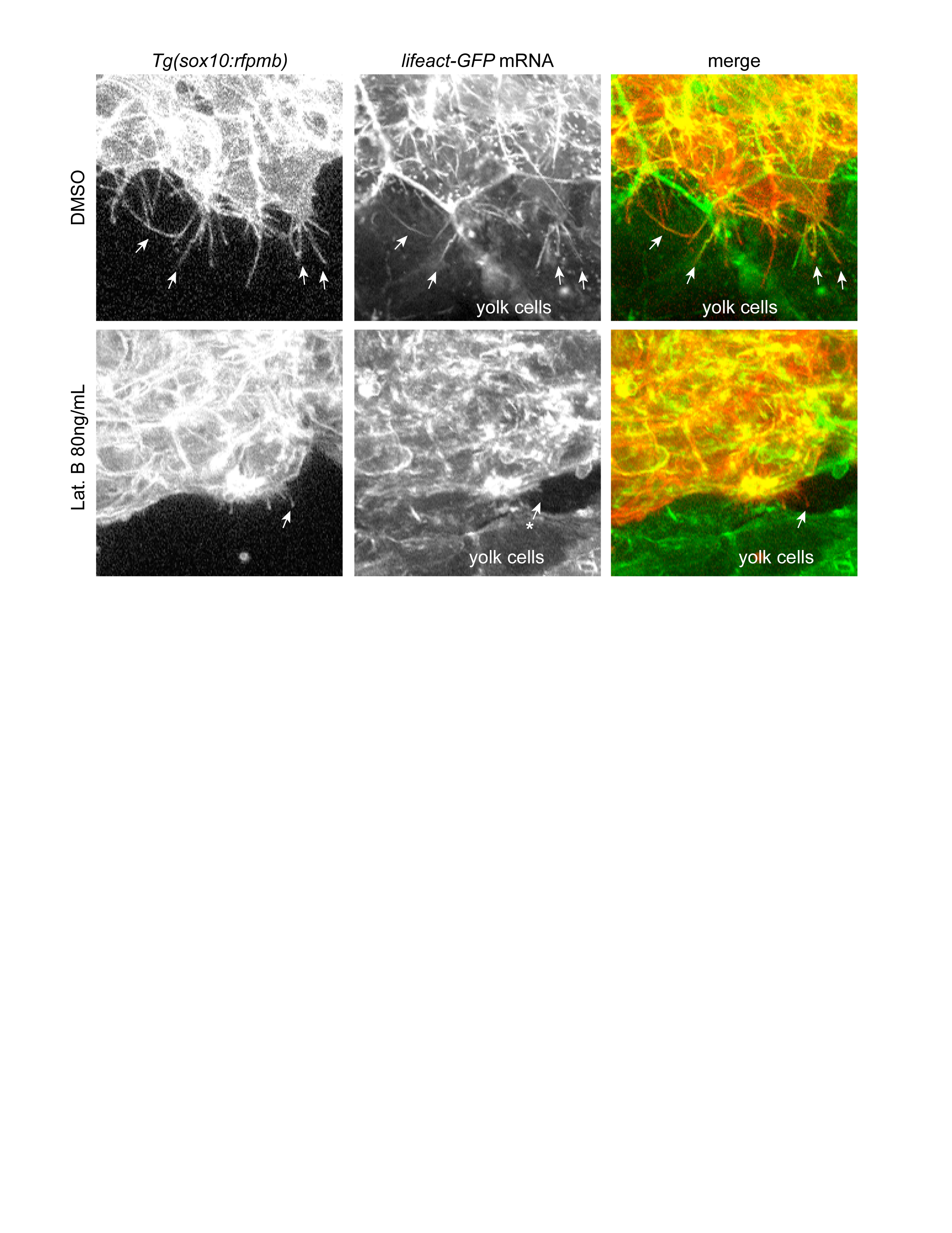

Supplement: S10 Fig — Representative maximum projection confocal images of the leading edge of second cranial NC stream from 26 hpf Tg(sox10:rfpmb) embryos injected with lifeact-GFP mRNA and treated with low dose Lat. B or DMSO. Arrows indicate filopodia. Asterisk denotes lack of Lifeact-GFP in RFP+ filopodia in Lat. B-treated embryo. F-actin in underlying yolk cells is also visible in all images. (TIF) [file pgen.1004946.s010.tif]
